# Rational Design of Cost-Effective 4-Styrylcoumarin Fluorescent Derivatives for Biomolecule Labeling

**DOI:** 10.3390/molecules28196822

**Published:** 2023-09-27

**Authors:** Raquel Eustáquio, João P. Prates Ramalho, Ana Teresa Caldeira, António Pereira

**Affiliations:** 1 HERCULES Laboratory, IN2PAST—Associate Laboratory for Research and Innovation in Heritage, Arts, Sustainability and Territory, University of Évora, Largo Marquês de Marialva 8, 7000-809 Évora, Portugal; raqueleustaquio98@hotmail.com (R.E.); atc@uevora.pt (A.T.C.); 2 Department of Chemistry and Biochemistry, School of Sciences and Technology, University of Évora, Rua Romão Ramalho 59, 7000-671 Évora, Portugal; jpcar@uevora.pt; 3 Associated Laboratory for Green Chemistry (LAQV) of the Network of Chemistry and Technology (REQUIMTE), University of Évora, Rua Romão Ramalho 59, 7000-671 Évora, Portugal; 4 City U Macau Chair in Sustainable Heritage, Sino-Portugal Joint Laboratory of Cultural Heritage Conservation Science, University of Évora, Largo Marquês de Marialva 8, 7000-809 Évora, Portugal

**Keywords:** fluorescent labels, coumarin, 4-styrylcoumarin derivatives, biomolecules

## Abstract

Fluorescent labels are key tools in a wide range of modern scientific applications, such as fluorescence microscopy, flow cytometry, histochemistry, direct and indirect immunochemistry, and fluorescence in situ hybridization (FISH). Small fluorescent labels have important practical advantages as they allow maximizing the fluorescence signal by binding multiple fluorophores to a single biomolecule. At present, the most widely used fluorescent labels available present small Stokes shifts and are too costly to be used in routine applications. In this work we present four new coumarin derivatives, as promising and inexpensive fluorescent labels for biomolecules, obtained through a cost-effective, efficient, and straightforward synthetic strategy. Density functional theory and time-dependent density functional theory calculations of the electronic ground and lowest-lying singlet excited states were carried out in order to gain insights into the observed photophysical properties.

## 1. Introduction

Fluorescent labels, also called fluorescent markers or probes, have become elementary in a broad range of modern scientific practices. The referred fluorophores provide fluorescence when it is absent or inadequate in molecules of interest in many applications, such as fluorescence microscopy, flow cytometry, histochemistry, direct and indirect immunochemistry, and fluorescence in situ hybridization (FISH) [1,2,3,4,5,6,7].

Generally, fluorescent labels are classified into three major groups, i.e., organic dyes, proteins, and nanoparticles, but the vast majority of fluorophores belong to the organic dyes group. Organic dyes have important practical advantages over the other dyes as they allow maximizing the fluorescence signal by binding multiple fluorophores to a single biomolecule, due to their small size. Also, these small fluorescent molecules can be chemically modified through simple and effective organic synthetic methods, which allows the synthesis of labels with a wide range of photophysical properties [8,9,10,11].

Considering the efficiency and sensitivity of the detection process, the photophysical properties of fluorescent labels (wavelength of maximum absorption, molar extinction coefficient, wavelength of maximum emission, Stokes shift, and fluorescence quantum yield) are determinant for the wide range of specific applications available [12].

The combination of fluorescence imaging with fluorophores based on organic molecules has become a powerful tool as it has enabled the understanding of biological events at the molecular level due to its high sensitivity or selectivity, non-invasive character, easy implementation, rapid response rate, real-time detection, spatiotemporal resolution, facile visualization, and in situ detection [13,14,15].

Due to the abundance of amino groups, or their easy incorporation into biomolecules, the amine-reactive fluorescent labels are most commonly used to prepare stable bioconjugates for a wide range of biological applications. However, for super-resolution multi-color imaging experiments, such as optical microscopy of biomolecules based on stimulated emission depletion (STED) and in Förster-type resonance energy transfer (FRET) applications, the development of new fluorescent labels with large Stokes shifts is essential and indispensable [16,17,18,19,20,21].

Currently, the most widely used fluorescent labels available present small Stokes shifts, many of which are under 30 nm and are too costly to be used in routine applications, which is their major handicap. In this context, coumarin derivatives can be a real solution to develop new cost-effective brightness fluorophores, with large Stokes shifts [12,22,23].

Coumarins, found in many plant species [24] and often classified as classical fluorescent dyes, have been modified to obtain coumarin-based fluorescent dyes with adjustable optical properties due to their excellent photophysical properties, good cell membrane permeability, nontoxicity, and cost-effectiveness [10,25,26]. Recent developments have conclusively shown that the arrangement of substituents and the types of substituents present in coumarin rings significantly impact the electronic delocalization within the conjugated π-system. Additionally, it is widely recognized that the inclusion of such substituents, forming donor-π-bridge-acceptor (D-π-A) structures, can facilitate an efficient intramolecular charge transfer (ICT) process. This, in turn, leads to remarkable and varied photophysical properties in coumarin derivatives [10,27,28,29,30,31,32,33,34]. Several of these coumarin derivatives have been applied in many important areas such as solar cells, organic light-emitting diodes, and fluorescent probes [26,35,36,37].

In this study, we aim to establish a cost-effective synthetic approach for producing four novel 4-styrylcoumarin derivatives (**9**, **14**, **15**, and **20**) with red-shifted fluorescence properties, utilizing the readily available and affordable 7-diethylamino-4-methylcoumarin (**1**) as our starting material. These newly synthesized compounds hold potential as fluorescent labels for biomolecules. To further enhance our understanding, we have conducted theoretical analyses on the optical, electronic, and geometric characteristics of these fluorescent labels in both their ground and lowest-energy singlet excited states. This analysis was performed using density functional theory (DFT) and time-dependent density functional theory (TD-DFT), complementing the overall investigation.

## 2. Results and Discussion

### 2.1. Synthesis and Characterization

**Figure 1:**
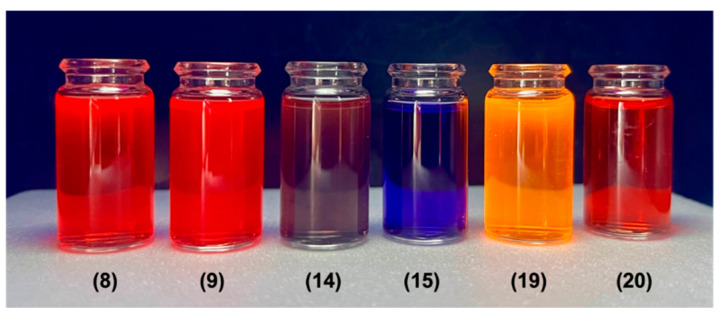
Photographic images of the synthesized fluorescent labels (**8**, **9**, **14**, **15**, **19**, and **20**) in MeCN at 365 nm.

**Scheme 1:**
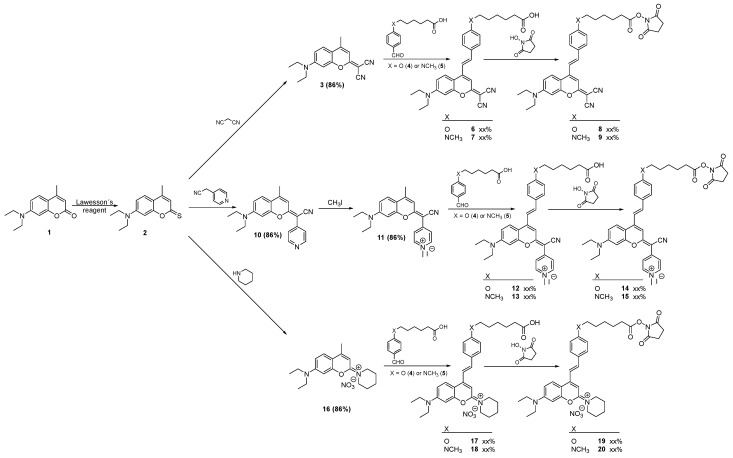
Synthetic route to the fluorescent labels **8**, **9**, **14**, **15**, **19**, and **20**.

The synthetic routes followed for the preparation of the fluorescent labels for biomolecules **8**, **9**, **14**, **15**, **19**, and **20** (Figure 1), using the inexpensive 7-diethylamino-4-methylcoumarin (**1**) as the starting material, are shown in Scheme 1. The main synthetic approach for producing 4-styrylcoumarin derivatives relied on the elevated acidity of the methyl protons found at position 4 within the coumarin ring. This acidity facilitated aldol condensation reactions, particularly when electron-withdrawing groups (EWGs) were introduced at position 2. We believed that introducing a 4-styryl group with electron-donating groups (EDGs) in the *para* position could enhance both the π-delocalization and the push–pull nature of the chromophore. These modifications can improve photophysical properties of the coumarin derivatives, such as high-fluorescence quantum yields, as well as higher bathochromic and large Stokes shifts.

The intermediates mentioned, which had electron-withdrawing groups (EWGs) at position 2, specifically **3**, **11**, and **16**, were readily synthesized with high yields (85% to 89%). This synthesis involved a thionation reaction on the carbonyl group of 7-diethylamino-4-methylcoumarin (**1**), followed by the condensation of the resulting thionated coumarin (**2**) with malononitrile, 4-pyridylacetonitrile hydrochloride, or piperidine, depending on the case.

The regioselective and highly efficient aldol condensation between intermediates **3**, **11**, and **16** and the aromatic aldehydes with EDGs at the *para* position (6-((4-formylphenyl)(methyl)amino)hexanoic acid and 6-(4-formylphenoxy)hexanoic acid) afforded the 4-styrylcoumarins **6**, **7**, **12**, **13**, **17**, and **18** in good to high yields.

The lower yields observed in the pyridinyl derivatives **12** and **13** may have been caused by an acidity decrease in the methyl protons present at position 4 due to the superior electronic delocalization across the molecular structure caused by the pyridine group.

The differences between the electronegativity of the nitrogen or oxygen atom in the electron-donating groups present in the aromatic aldehydes may explain the small difference in reaction yields observed, with a small advantage for the amino aldehydes.

The esterification of coumarin carboxylic acids **6**, **7**, **12**, **13**, **17**, and **18** produced the *N*-hydroxysuccinimide (NHS) esters **8**, **9**, **14**, **15**, **19**, and **20** as promising reactive fluorescent labels for biomolecules, in high yields. The referred fluorescent labels can be obtained through four effective and linear synthetic steps from cheap commercially available precursors (five steps to **14** and **15**). All new compounds (**9**, **14**, **15**, and **20**) were fully characterized by 1D and 2D NMR spectroscopy and HRMS. The spectral data were consistent with the proposed structures.

### 2.2. Photophysical Characterization

**Figure 2:**
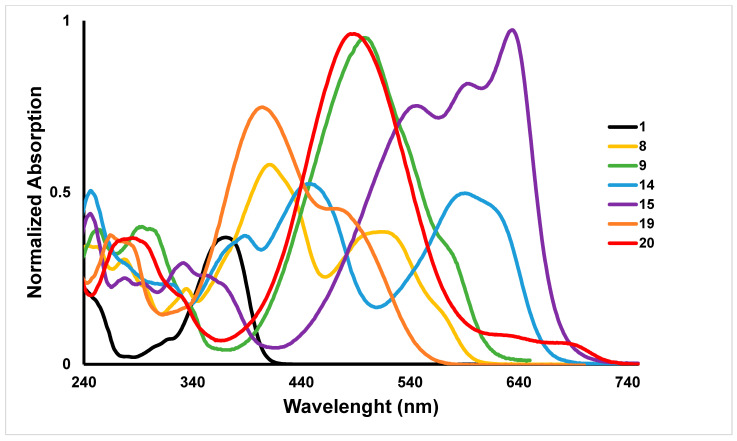
UV/Vis spectra of 7-diethylamino-4-methylcoumarin (**1**) and the synthesized fluorescent labels (**8**, **9**, **14**, **15**, **19**, and **20**) in MeCN.

We conducted a study on the photophysics of the newly synthesized coumarin derivatives, examining their absorption and emission characteristics, along with fluorescence quantum yields. Table 1 provides a summary of these properties, while Figure 2 illustrates the UV/Vis spectra of these coumarin derivatives. The inclusion of electron-withdrawing groups (EWGs) at position 2, in the coumarin ring, promoted effective bathochromic shifts of 66, 106, and 179 nm, in the intermediates **16**, **3**, and **11**, respectively. These modifications increased the push–pull character from the electron-donating group NEt_2_, at position 7, by extending the π-system with the introduction of a new double bond that was conjugated with the aforementioned electron-withdrawing groups (EWGs). The combination between an effective positive charge and the electronic delocalization present in the pyridine moiety of the EWG intermediate **11** allowed the largest bathochromic shift observed. In contrast, the intermediate **16** that only presented a positive charge as EWG had the lowest bathochromic shift value.

Unsurprisingly, these shifts were further increased to longer wavelengths with the inclusion of a 4-styryl moiety, containing one electron-donating nitrogen or oxygen group at the *para* position. As expected, the nitrogen group, when compared with the oxygen group, promoted higher bathochromic shifts (approximately more than 40%) due to the superior mesomeric electron-donating character of this substituent.

The molar absorption coefficients of the fluorescent labels (**8**, **9**, **14**, **15**, **19**, and **20**) were also strongly influenced by the nature of the EWGs at position 2 and the EDGs at position 4 in the coumarin ring (e.g., ε (**8**) = 24,000 cm^−1^ M^−1^ vs. ε (**15**) = 113,500 cm^−1^ M^−1^). Considering only the 4-styryl moiety effect, the presence of the nitrogen group more than doubled the molar absorption coefficients when compared with the counterpart oxygen group.

The new fluorescent labels **14** and **15** have high molar absorption coefficients, approximately twice as much as the others, which may be due to the referred combination present in the pyridine moiety of the EWG.

All fluorescent labels exhibited large Stokes shifts, which were essential to the effective intramolecular charge transfer (ICT) process of the emissive excited state, due to the extension of the π-conjugated system in the molecule. Large Stokes shifts minimized the undesirable spectral overlap between absorption and emission, allowing the reduction in the interference and the quenching process, providing an effective detection of the fluorescence emission.

The decrease in fluorescence quantum yields in the fluorescent labels **14** and **15** could be related to the effective intramolecular charge transfer process due to the increase in the extension of π-conjugated system in these molecules [38]. Obviously, this effect was not so pronounced in the fluorescent labels **19** and **20**, and that was why they had high fluorescence quantum yields. In agreement with the above, due to the lower electron-donating mesomeric character of the oxygen group in the 4-stryl moiety, compared with the nitrogen group, this substituent promoted higher-fluorescence quantum yields.

### 2.3. Theoretical Calculations

**Figure 3:**
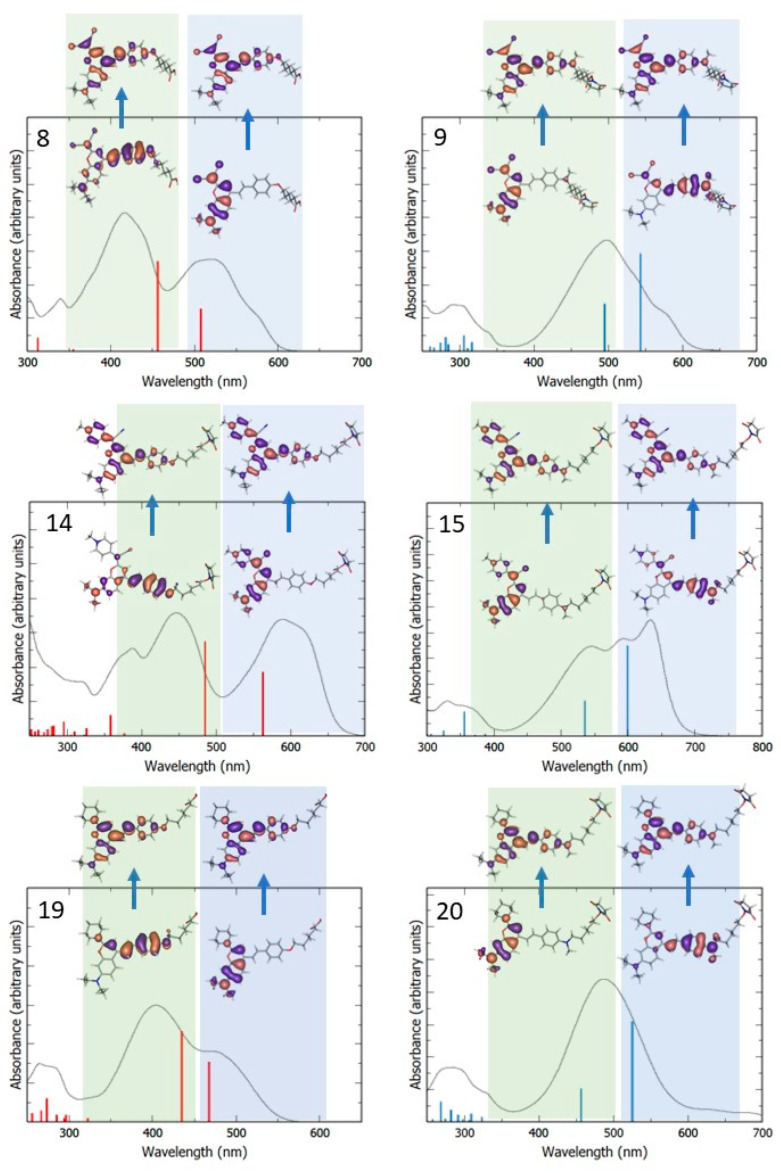
Experimental spectra (gray) compared with the calculate excitations (red and blue) and the molecular orbitals involved in the two lowest-energy electronic transitions of the **8**, **9**, **14**, **15**, **19**, and **20** dyes in MeCN.

The lower-energy bands of the spectra were dominated by two excitations, S_1_ and S_2_, as can be seen in Figure 3. The lowest-energy excitations S_1_ and S_2_ consisted mainly of HOMO → LUMO and HOMO-1 → LUMO one-electron contributions, respectively, for all the studied compounds, and their relative oscillator strengths depended on the compound possessing the oxygen or the nitrogen group (Table S1). For the oxygen group compounds **8**, **14**, and **19**, the S_2_ excitation was dominant, while the S_1_ was the most intense for the nitrogen group compounds **9**, **15**, and **20**. This accounted for the elevated molar absorption coefficients observed in the nitrogen group compounds in comparison with their oxygen group counterparts. Additionally, the higher oscillator strength value of compound **15** aligned with the greater molar absorption observed for this particular compound.

The shape and spatial distribution of the HOMO, HOMO-1, and LUMO states exhibited notable differences, indicating distinct characteristics of the intramolecular charge transfer that took place upon excitation. For all the compounds, neither the HOMO, the HOMO-1, nor the LUMO orbitals presented relevant contributions over the attached reactive group, indicating that it did not take part in the π-conjugation framework and did not interfere in the excitation process. This is an important feature since a minimal interference of the attachment of the dye labels to a biomolecule with their fluorescence properties is desirable.

Turning to the HOMO states, the main feature that emerged was that the HOMO orbitals were mostly localized in the coumarin for the oxygen group compounds, while for the nitrogen group compounds, they were located on the 4-styryl group. Conversely, the inverse occurred for the HOMO-1 orbitals, with the HOMO of the oxygen group compounds presenting great similarity with the HOMO-1 of the nitrogen group compounds and vice versa.

For all compounds, however, the LUMO orbitals showed less localization and extended over the bridging zone and did not present important differences between the two compound families. This extension facilitated low-energy internal charge transfer absorptions. The most intense transition, whether being that with the longest wavelength or not, corresponded in all cases to a transition from the state mainly located in the styryl group to the LUMO, which suggested those were the states that overlapped to a larger extent with the LUMO orbitals. The lowest-energy transition in compounds **9**, **15**, and **20** exhibiting the highest oscillator strength involved a charge transfer process from the 4-styryl group to the coumarin moiety, highlighting the significant donor ability of the amine group connected to the 4-styryl moiety.

The Δ**r** index [39], a measure of the charge transfer length, was employed to characterize the nature of electronic transitions. Transitions exhibiting a substantial Δ**r** index indicated a significant charge transfer (CT) character, with a commonly accepted threshold of 2.0 Å used as a criterion for assigning the CT character. Following this criterion, most of the S_1_ and S_2_ excitations could be classified as CT. The smaller values arose in derivative **8**, and particularly high values of Δ**r** occurred for compound **19**, indicating a large distance displacement of the charge upon excitation.

**Figure 4:**
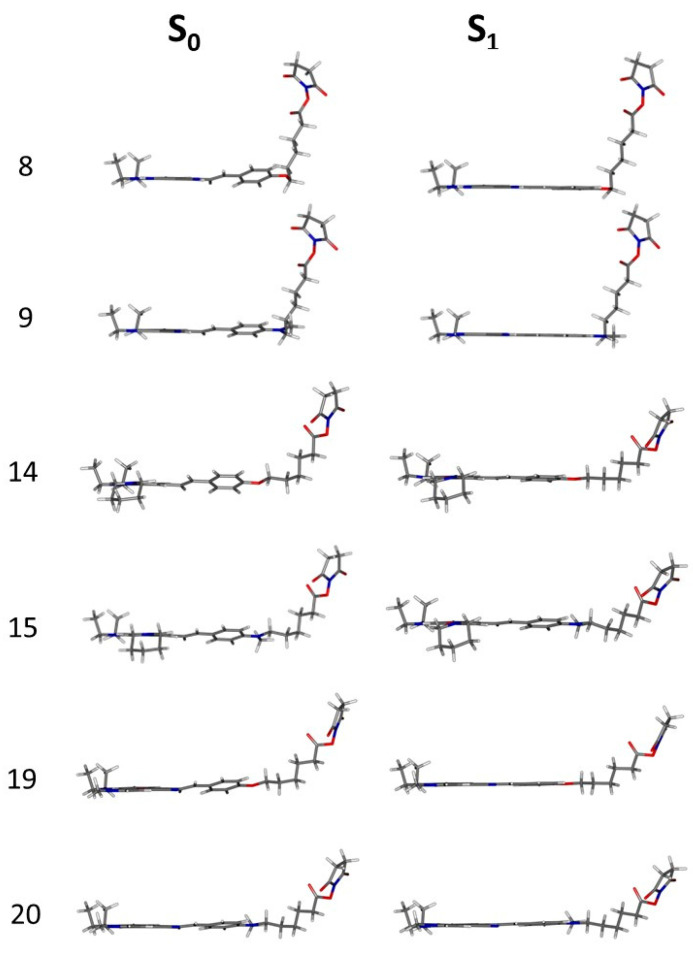
Optimized molecular geometries computed at the PBE0/6-31G (d,p) level in MeCN of the coumarin derivatives, in both the ground and the first excited singlet states.

Figure 4 presents the optimized molecular geometry of the coumarin derivatives in both the ground and the first excited singlet states. In the ground state, all the coumarin derivatives exhibited a deviation from planarity between the average plane of the coumarin ring moiety and the plane of the 4-styryl group. This deviation limited the π-conjugation that connected the donor and acceptor groups within the molecules. The magnitude of this dihedral angle varied among the compounds with derivative **15** presenting the smaller dihedral angle of 8.4° and compound **19** presenting the higher dihedral angles of 19.9° (Table S2 and Figure S28).

In contrast, the S_1_ electronic excited state revealed a distinct scenario, where all the molecules became nearly planar. The deviations from planarity were minimal, ranging from a minimum of 0.1° for compound **9** to a maximum of 7.4° for compound **20**. The transition from the S_0_ state to the S_1_ state was also characterized by a decrease in the bond length alternation (BLA), the average length difference between a single and adjacent double bond, which considerably reduces, for all compounds, in the excited S_1_ state. In the S_0_ state the compounds exhibited variations of BLA ranging from 0.08 Å for compounds **9** and **20** to 0.10 Å for compounds **8** and **15**. However, in the excited state S_1_, the BLA significantly decreased to 0.04 Å (compounds **8**, **9**, and **19**) to a maximum of 0.06 Å for dye **20**.

According to Kasha’s rule [40], fluorescence in most cases is restricted to the lowest-lying excited state with the higher electronic excited states typically not directly contributing to the emission of excited fluorophores. Therefore, the emissions of the studied dyes would arise from the S_1_ state.

The emission properties of the dyes were calculated from the optimized S_1_ state geometry and are depicted in Table S3. The emission wavelengths S_1_ → S_0_ were systematically smaller than those corresponding to the absorption S_0_ → S_1_ process based on the ground state optimized geometry. The observed geometry changes that occurred in the electronic excited state S_1_ promoted enhanced electronic delocalization within the π-conjugation structure, resulting in a reduction in the HOMO and LUMO energy gap. Consequently, following a vertical excitation, there was a notable structural relaxation of the excited state that occurred before emission [41,42], which led to a smaller emission energy compared with the excitation energy, thus resulting in a substantial Stokes shift.

From the lowest-lying excited state S_1_ geometry optimization performed, the theoretical fluorescent lifetime of the excited states can be calculated from Einstein’s transition probabilities of the spontaneous transitions equation

$$\tau =\frac{c^{3}}{2E^{2}f}$$

where *τ* is the fluorescent lifetime, *c* is the velocity of light in vacuum, *E* is the transition energy, and *f* is the corresponding oscillator strength of the S_1_→S_0_ transition. The fluorescent lifetimes of the calculated fluorescent dyes are depicted in Table S3, and for all dyes, all the values were shorter than 10 ns, typical for emissive states of organic fluorophores, while larger radiative lifetimes corresponded to nonradiative states. However, since nitrogen compounds exhibited a greater oscillator strength in the S_1_ → S_0_ transition, their fluorescent lifetimes were shorter compared with their oxygen counterparts.

## 3. Experimental Section

### 3.1. Materials and Equipment

Analytical grade starting materials and reagents were used, purchased from Aldrich (St. Louis, MO, USA). Organic solvents were dried over appropriate drying agents and distilled before use. UV-Vis absorption spectra were recorded using a Thermo Electron Spectrophotometer Corporation (Waltham, MA, USA), model Nicolet Evolution 300. Fluorescence spectra were recorded using a PerkinElmer Model LS 55 spectrophotometer (PerkinElmer, Waltham, MA, USA). Emission spectra were collected with a 5.0 nm slit bandwidth for both excitation and emission, with correction files. All spectroscopic measurements were performed in 3 mL quartz fluorescence cuvettes with a 1 cm optical path at a temperature of 21 °C. Acetonitrile (CH_3_CN) was used as the solvent for these measurements. FTMS-ESI mass spectra were obtained using a Thermo Scientific Q Exactive Orbitrap Mass Spectrometer (Thermo Scientific, Waltham, MA, USA). Nuclear magnetic resonance (NMR) spectra were recorded at 400 MHz for ^1^H NMR and 100 MHz for ^13^C NMR, using a Brucker Advance III spectrometer (Billerica, MA, USA). Deuterated chloroform (CDCl_3_), deuterated methanol (CD_3_OD), or deuterated dimethyl sulfoxide (CD_3_)_2_SO was used as the solvent for NMR experiments. The chemical shift (δ) was measured in parts per million (ppm), coupling constants (J) were provided in Hertz (Hz), relative intensity was indicated by the number of protons (H), and multiplicities were indicated by singlet (s), doublet (d), triplet (t), quadruplet (q), and multiplet (m).

### 3.2. Synthesis

#### 3.2.1. Synthesis of 2-(7-(Diethylamino)-4-methyl-2H-chromen-2-ylidene)malononitrile (**3**)

The synthesis of dicyanomethylenecoumarinmethyl derivative (**3**) was carried out following the procedure outlined by Gandioso et al. [33].

#### 3.2.2. Synthesis of 6-(4-Formylphenoxy)hexanoic Acid (**4**)

Synthesis of 6-(4-formylphenoxy)hexanoic acid derivative (**4**) was performed according to the method described by Eustáquio et al. [27].

#### 3.2.3. Synthesis of 6-((4-Formylphenyl)(methyl)amino)hexanoic Acid (**5**)

For the synthesis of ethyl 6-(methyl(phenyl)amino)hexanoate, a mixture of *N*-methylaniline (1.0 g, 18.6 mmol, 1.0 eq), ethyl 6-bromohexanoate (3.32 mL, 18.6 mmol, 1.0 eq), and potassium carbonate (2.58 g, 18.6 mmol, 1.0 eq) in dry acetonitrile (20 mL) was stirred at 85 °C, for a period of 24 h. The reaction was continuously monitored by TLC, using CH_2_Cl_2_/hexane (5:5) as the eluent. The reaction mixture was evaporated to dryness, and the residue was purified by flash chromatography, using a hexane, CH_2_Cl_2_/hexane (5:5), and CH_2_Cl_2_ as eluents, to yield ethyl 6-(methyl(phenyl)amino)hexanoate, as a colorless oil (3.95 g, 85%).

For the synthesis of ethyl 6-((4-formylphenyl)(methyl)amino)hexanoate, a mixture of DMF (2.1 mL, 26.7 mmol, 3.6 eq) and POCl_3_ (700 µL, 7.42 mmol, 1.0 eq) in an ice bath, was stirred for 5 min. The ice bath was removed, and the ethyl 6-(methyl(phenyl)amino)hexanoate (1.85 g, 7.42 mmol, 1.0 eq) was added. The reaction mixture was stirred at 100 °C, for 2 h. To the reaction mixture at room temperature, 2 mL of a NaOAc (7M) solution was added, keeping the stirring for 2 h. The reaction was continuously monitored by TLC, using CH_2_Cl_2_ as eluent. The reaction mixture was evaporated to dryness, and the residue was purified by flash chromatography, using CH_2_Cl_2_ as eluent, to yield ethyl 6-((4-formylphenyl)(methyl)amino)hexanoate, as a white solid (1.50 g, 73%).

A mixture of ethyl 6-((4-formylphenyl)(methyl)amino) hexanoate (1.3 g, 4.69 mmol, 1.0 eq) and 2 M NaOH solution (11.7 mL, 23.4 mmol, 5.0 eq) in THF (20 mL) was stirred at room temperature, for 24 h. The reaction mixture was then acidified with 6 M HCl and was continuously monitored by TLC, using CHCl_3_/CH_3_OH (9:1) as the eluent. The reaction mixture was evaporated to dryness, and the residue was purified by flash chromatography, using CHCl_3_/CH_3_OH (95:5) and CHCl_3_/CH_3_OH (9:1) as eluents, to yield 6-((4-formylphenyl)(methyl)amino)hexanoic acid, as a slightly yellow solid (1.15 g, 98%).

The ^1^H NMR (400 MHz, CDCl_3_, δ, ppm) values were as follows: 1.39–1.45 (2H, m, H-4), 1.62–1.70 (4H, m, H-3, H-5), 2.38 (2H, t, *J* = 7.3, H-2), 3.04 (3H, s, NCH_3_), 3.41 (2H, t, *J* = 7.5, H-6), 6.67 (2H, d, *J* = 8.8, H-8, H-12), 7.72 (2H, d, *J* = 8.8, H-9, H-11), and 9.70 (1H, s, C*H*O). The ^13^C NMR (100 MHz, CDCl_3_, δ, ppm) values were as follows: 24.6 (C-3), 26.5 (C-4), 26.7 (C-5), 34.0 (N*C*H_3_), 52.4 (C-6), 111.0 (C-8, C-12), 125.0 (C-10), 132.4 (C-9, C11), 153.6 (C-7), 179.3 (*C*OOH), and 190.5 (*C*HO).

#### 3.2.4. Synthesis of (*E*)-6-(4-(2-(2-(Dicyanomethylene)-7-(diethylamino)-2H-chromen-4-il)vinyl)phenoxy)hexanoic Acid (**6**)

Synthesis of (*E*)-6-(4-(2-(2-(dicyanomethylene)-7-(diethylamino)-2*H*-chromen-4-yl)vinyl)phenoxy)hexanoic acid (**6**) was performed according to the method described by Eustáquio et al. [27].

#### 3.2.5. Synthesis of (*E*)-6-((4-(2-(2-(Dicyanomethylene)-7-(diethylamino)-2H-chromen-4yl)vinyl)phenyl)(methyl) amino)hexanoic Acid (**7**)

A mixture of compound **3** (175 mg, 0.626 mmol, 1.0 eq), 6-((4-formylphenyl)(methyl)amino)hexanoic acid **5** (156 mg, 0.626 mmol, 1.0 eq), and piperidine (124 µL, 1.25 mmol, 2.0 eq) in dry acetonitrile (10 mL) was stirred at 85 °C, for a period of 72 h. The reaction mixture, at room temperature, was then acidified with 6 M HCl and was continuously monitored by TLC, using eluent CHCl_3_/CH_3_OH/H_2_O (65:10:1) as the eluent. The reaction mixture was evaporated to dryness, and the residue was purified by flash chromatography, using CH_2_Cl_2_ and CHCl_3_/CH_3_OH (99:1) as eluents, to yield (*E*)-6-((4-(2-(2-(dicyanomethylene)-7-(diethylamino)-2*H*-chromen-4yl)vinyl)phenyl)(methyl)-amino)hexanoic acid, as an orange solid (273 mg, 86%).

The ^1^H NMR (400 MHz, CDCl_3_, δ, ppm) values were as follows: 1.25 (6H, t, *J* = 7.1, N(CH_2_*CH*_3_)_2_), 1.36–1.46 (2H, m, H-26), 1.61–1.74 (4H, m, H-25, H-27), 2.39 (2H, t, *J* = 7.3, H-28), 3.02 (3H, s, H-30), 3.39 (2H, t, *J* = 7.5, H-24), 3.45 (4H, q, *J* = 7.1, N(*CH*_2_CH_3_)_2_), 6.55 (1H, d, *J* = 2.6, H-8), 6.67 (2H, d, *J*_20,19_ = 8.9, H-20, H-22), 6.70 (1H, dd, *J*_6,5_ = 9.3, *J*_6,8_ = 2.6, H-6), 6.80 (1H, s, H-3), 7.09 (1H, d, *J*_9,17_ = 15.8, H-9), 7.40 (1H, d, *J*_17,9_ = 15.8, H-17), 7.48 (2H, d, *J*_19,20_ = 8.9, H-19, H-23), and 7.67 (1H, d, *J*_5,6_ = 9.3, H-5). The ^13^C NMR (100 MHz, CDCl_3_, δ, ppm) values were as follows: 12.7 (C-11, C-13), 24.6 (C-27), 26.6 (C-26), 26.8 (C-25), 33.9 (C-28), 38.6 (C-30), 45.0 (C-10, C-12), 52.4 (C-24), 97.7 (C-8), 100.9 (C-3), 108.9 (C-4a), 110.5 (C-6), 112.9 (C-14, C-20, C-22), 113.0 (C-9), 115.7 (CN), 116.7 (CN), 123.1 (C-18), 125.7 (C-5), 130.1 (C-19, C-23), 140.0 (C-17), 147.3 (C-4), 150.8 (C-21), 151.5 (C-7), 155.6 (C-8a), 171.3 (C-2), and 178.7 (C-29). The UV λ^max^ (CH_3_CN, nm) values were as follows: 500 and 578. The FTMS(+) calc. values for C_31_H_34_O_3_N_4_ [M + H]^+^ were 511.2704 and 511.2696. The UV λ^max^ (nm, CH_3_CN) values were as follows: 500 and 578. The ε (cm^−1^ M^−1^) values were as follows: 59,000. Φ_F_ = 0.27.

#### 3.2.6. Synthesis of (*E*)-2,5-Dioxopyrrolidin-1-il 6-(4-(2-(2-(dicyanomethylene)-7-(diethylamino)-2H-chromen-4-yl)vinyl)phenoxy)hexanoate (**8**)

The synthesis of (*E*)-2,5-dioxopyrrolidin-1-il 6-(4-(2-(2-(dicyanomethylene)-7-(diethylamino)-2*H*-chromen-4-il)vinyl)phenoxy)hexanoate (**8**) was performed according to the method described by Eustáquio et al. [27].

#### 3.2.7. Synthesis of (*E*)-2,5-Dioxopyrrolidin-1-yl 6-((4-(2-(2-(dicyanomethylene)-7-(diethylamino)-2H-chromen-4-yl) vinyl)phenyl)(methyl)amino)hexanoate (**9**)

A mixture of compound **7** (150 mg, 0.294 mmol, 1.0 eq), DCC (73 mg, 0.352 mmol, 1.2 eq), and DMAP (0.32 mg, 2.64 × 10^−3^ mmol, 10%) in dry DMF (2 mL) and dry acetonitrile (8 mL) was stirred at room temperature for 5 min. After this reaction time, *N*-hydroxysuccinimide (41 mg, 0.352 mmol, 1.2 eq) was added, and the reaction was stirred at room temperature for 5 h. The reaction was monitored by TLC, using CH_2_Cl_2_ as the eluent. The reaction mixture was evaporated to dryness, and the residue was purified by flash chromatography using CH_2_Cl_2_ and CH_2_Cl_2_/CH_3_OH (95:5) as eluents, to yield (*E*)-6-((4-(2-(2-(dicyanomethylene)-7-(diethylamino)-2*H*-chromen-4-yl)vinyl)phenyl)(methyl)-amino)hexanoate, as an orange solid (170 mg, 95%).

The ^1^H NMR (400 MHz, CDCl_3,_ δ, ppm) values were as follows: 1.25 (6H, t, *J* = 7.1, N(CH_2_*CH*_3_)_2_), 1.45–1.49 (2H, m, H-26), 1.63–1.68 (2H, m, H-25), 1.77–1.82 (2H, m, H-27), 2.63 (2H, t, *J* = 7.3, H-28), 2.84–2.85 (4H, m, H-32, H-33), 3.03 (3H, s, H-30), 4.21 (2H, t, *J* = 7.5, H-24), 3.45 (4H, q, *J* = 7.1, N(*CH*_2_CH_3_)_2_), 6.58 (1H, d, *J* = 2.6, H-8), 6.69 (2H, d, *J*_20,19_ = 8.8, H-20, H-22), 6.69 (1H, dd, *J*_6,5_ = 9.3, *J*_6,8_ = 2.6, H-6), 6.84 (1H, s, H-3), 7.11 (1H, d, *J*_9,17_ = 15.8, H-9), 7.43 (1H, d, *J*_17,9_ = 15.8, H-17), 7.50 (2H, d, *J*_19,20_ = 8.8, H-19, H-23), and 7.68 (1H, d, *J*_5,6_ = 9.3, H-5). The ^13^C NMR (100 MHz, CDCl_3,_ δ, ppm) values were as follows: 12.7 (C-11, C-13), 24.6 (C-27), 25.7 (C-32, C-33), 26.3 (C-26), 26.6 (C-25), 31.0 (C-28), 38.3 (C-30), 45.0 (C-10, C-12), 52.5 (C-24), 97.7 (C-8), 101.1 (C-3), 109.0 (C-4a), 110.4 (C-6), 112.9 (C-14, C-20, C-22), 113.3 (C-9), 115.6 (CN), 116.6 (CN), 123.1 (C-18), 125.7 (C-5), 130.0 (C-19, C-23), 140.0 (C-17), 147.3 (C-4), 150.8 (C-21), 151.5 (C-7), 155.6 (C-8a), 168.6 (C-2), 169.3 (C-31, C-34), and 171.4 (C-29). The FTMS(+) calc. values for C_35_H_38_O_5_N_5_ [M + H]^+^ were 608.2868 and 608.2863. The UV λ^max^ (nm, CH_3_CN) values were as follows: 498, 576. The ε (cm^−1^ M^−1^) values were as follows: 59,000. Φ_F_ = 0.27.

#### 3.2.8. Synthesis of (*E*)-2-(7-(Diethylamino)-4-methyl-2H-chromen-2-ylidene)-2-(pyridin-4-yl)acetonitrile (**10**)

The synthesis of (*E*)-2-(7-(diethylamino)-4-methyl-2*H*-chromen-2-ylidene)-2-(pyridin-4-yl)acetonitrile (**10**) was performed according to the methods described by Gandioso et al. [34].

#### 3.2.9. Synthesis of (*E*)-4-(Cyano(7-(diethylamino)-4-methyl-2H-chromen-2-ylidene)methyl)-1-methylpyridin-1-ium Iodide (**11**)

A mixture of compound **10** (300 mg, 0.905 mmol, 1.0 eq) and iodomethane (141 µL, 2.24 mmol, 2.5 eq) in CHCl_3_ (20mL) was stirred at room temperature overnight. The reaction was continuously monitored by TLC, using CHCl_3_/CH_3_OH (95:5) as the eluent. The reaction mixture was evaporated to dryness and the residue purified by flash chromatography, using CHCl_3_/CH_3_OH (95:5), CHCl_3_/CH_3_OH (9:1), CHCl_3_/CH_3_OH (8:2), and CHCl_3_/CH_3_OH/H_2_O (65:10:1) as eluents, to yield (*E*)-4-(cyano(7-(diethylamino)-4-methyl-2*H*-chromen-2-ylidene)methyl)-1-methyl pyridin-1-ium iodide, as a purple solid (400 mg, 93%).

The ^1^H NMR (400 MHz, (CD_3_)_2_SO, δ, ppm) values were as follows: 1.17 (6H, t, *J* = 7.0, N(CH_2_*CH*_3_)_2_), 2.49 (3H, s, H-9), 3.54 (4H, q, *J* = 7.0, N(*CH*_2_CH_3_)_2_), 4.18 (3H, s, H-21), 6.80 (1H, d, *J*_3,9_ = 7.2, H-3), 6.91 (2H, m, H-6, H-8), 7.64 (1H, dd, *J*_5,9_ = 5.2, *J*_5,6_ = 9.2, H-5), 8.07 (2H, m, H-17, H-20), and 8.58 (2H, d, *J*_18,17_ = 6.7, H-18, H-19). The ^13^C NMR (100 MHz, (CD_3_)_2_SO, δ, ppm) values were as follows: 12.5 (C-11, C-13), 18.5 (C-9), 44.3 (C-10, C-12), 46.1 (C-21), 78.0 (C-16), 96.4 (C-8), 110.2 (C-3), 110.3 (C-4a), 111.7 (C-6), 118.2 (C-15), 120.6 (C-17, C-20), 127.0 (C-5), 143.8 (C-18, C-19), 148.4 (C-14), 151.9 (C-7), 152.2 (C-4), 154.7 (C-8a), and 166.5 (C-2). The FTMS(+) calc. values for C_22_H_24_ON_3_ [M-(I^-^)]^+^ were 346.1914 and 346.1906. The UV λ^max^ (nm, CH_3_CN) value was 550. The ε (cm^−1^ M^−1^) value was 80,000. Φ_F_ = 0.36.

#### 3.2.10. Synthesis of 4-(((*E*)-4-((*E*)-4-((5-Carboxypentyl)oxy)styryl)-7-(diethylamino)-2H-chromen-2-ylidene)(cyano)methyl)-1-methylpyridin-1-ium Iodide (**12**)

A mixture of compound **11** (300 mg, 0.634 mmol, 1.0 eq), 6-(4-formylphenoxy)hexanoic acid **4** (150 mg, 0.634 mmol, 1.0 eq), and piperidine (126 µL, 1.27 mmol, 2.0 eq) in dry acetonitrile (4 mL) was stirred at 85 °C, for a period of 48 h. The reaction mixture, at room temperature, was then acidified with 6M HCl and was continuously monitored by TLC, using CHCl_3_/CH_3_OH/H_2_O (65:10:1) as the eluent. The reaction mixture was evaporated to dryness, and the residue was purified by flash chromatography, using CHCl_3_/CH_3_OH/H_2_O (65:10:1) as the eluent, to yield 4-(((*E*)-4-((*E*)-4-((5-carboxypentyl)oxy)styryl)-7-(diethylamino)-2*H*-chromen-2-ylidene)(cyano)methyl)-1-methylpyridin-1-ium iodide, as a dark green solid (312 mg, 72%).

The ^1^H NMR (400 MHz, (CD_3_)_2_SO, δ, ppm) values were as follows: 1.16 (6H, t, *J* = 6.9, N(CH_2_*CH*_3_)_2_), 1.45 (2H, m, H-31), 1.58 (2H, m, H-32), 1.74 (2H, m, H-30), 2.24 (2H, t, H-33), 3.51 (4H, q, *J* = 6.9, N(*CH*_2_CH_3_)_2_), 4.03 (2H, t, H-29), 4.13 (3H, s, H-21), 6.84 (1H, sl, H-6), 6.85 (1H, sl, H-8), 6.98 (1H, s, H-3), 6.99 (2H, d, *J*_25,24_ = 8.6, H-25, H-27), 7.54 (2H, s, H-9, H-22), 7.78 (2H, *J*_24,25_ = 8.6, H-24, H-28), 7.96 (2H, d, *J*_17,18_ = 7.0, H-17, H-20), 8.07 (1H, d, *J*_5,6_ = 9.7, H-5), and 8.47 (2H, d, *J*_18,17_ = 7.0, H-18, H-19). The ^13^C NMR (100 MHz, (CD_3_)_2_SO, δ, ppm) values were as follows: 12.5 (C-11, C-13), 24.3 (C-32), 25.2 (C-31), 28.4 (C-30), 33.8 (C-33), 44.2 (C-10, C-12), 45.8 (C-21), 67.6 (C-29), 77.7 (C-16), 96.7 (C-8), 103.3 (C-3), 108.6 (C-4a), 111.6 (C-6), 114.8 (C-25, C-27), 117.2 (C-9), 118.5 (C-15), 119.9 (C-17, C-20), 127.0 (C-5), 128.1 (C-23), 130.4 (C-24, C-28), 139.1 (C-22), 143.3 (C-18, C-19), 147.7 (C-4), 148.4 (C-14), 151.9 (C-7), 155.1 (C-8a), 160.5 (C-26), 166.0 (C-2), and 174.5 (C-34). The FTMS(+) calc. values for C_35_H_38_O_4_N_3_ [M-(I^-^)]^+^ were 564.28568 and 564.2849. The UV λ^max^ (nm, CH_3_CN) values were as follows: 593 and 615. The ε (cm^−1^ M^−1^) value was 55,000. Φ_F_ = 0.07.

#### 3.2.11. Synthesis of 4-(((*E*)-4-((*E*)-4-((5-Carboxypentyl)(methyl)amino)styryl)-7-(diethylamino)-2H-chromen-2-ylidene)(cyano)methyl)-1-methylpyridin-1-ium Iodide (**13**)

A mixture of compound **11** (150 mg, 0.314 mmol, 1.0 eq), 6-((4-formylphenyl)(methyl)amino)hexanoic acid **5** (79 mg, 0.314 mmol, 1.0 eq), and piperidine (31 µL, 0.314 mmol, 1.0 eq) in dry acetonitrile (10 mL) was stirred at 85 °C, for a period of 24 h. The reaction mixture, at room temperature, was then acidified with 6M HCl and was continuously monitored by TLC, using CHCl_3_/CH_3_OH/H_2_O (65:10:1) as the eluent. The reaction mixture was evaporated to dryness, and the residue was purified by flash chromatography, using CHCl_3_/CH_3_OH/H_2_O (65:10:1) and CHCl_3_/CH_3_OH/H_2_O (65:20:2) as eluents, to yield 4-(((*E*)-4-((*E*)-4-((5-carboxypentyl)(methyl)amino)styryl)-7-(diethylamino)-2*H*-chromen-2-ylidene)(cyano)methyl)-1-methylpyridin-1-ium iodide, as a dark blue solid (168 mg, 75%).

The ^1^H NMR (400 MHz, (CD_3_)_2_SO, δ, ppm) values were as follows: 1.14 (6H, t, *J* = 6.9, N(CH_2_*CH*_3_)_2_), 1.27–1.33 (2H, m, H-31), 1.48–1.56 (4H, m, H-30, H-32), 2.17 (2H, t, *J* = 7.3 H-33), 2.99 (3H, s, H-35), 3.40 (2H, t, *J* = 7.0, H-29), 3.45 (4H, q, *J* = 6.9, N(*CH*_2_CH_3_)_2_), 4.05 (3H, s, H-21), 6.68 (2H, d, *J*_25,24_ = 8.8, H-25, H-27), 6.72 (1H, sl, H-8), 6.75 (1H, d, *J*_6,5_ = 9.3, H-6), 6.82 (1H, s, H-3), 7.27 (1H, d, *J*_9,22_ = 15.6, H-9), 7.38 (1H, d, *J*_22,9_ = 15.6, H-22), 7.59 (2H, *J*_24,25_ = 8.8, H-24, H-28), 7.79 (2H, d, *J*_17,18_ = 7.1, H-17, H-20), 7.97 (1H, d, *J*_5,6_ = 9.3, H-5), and 8.36 (2H, d, *J*_18,17_ = 7.1, H-18, H-19). The ^13^C NMR (100 MHz, (CD_3_)_2_SO, δ, ppm) values were as follows: 12.5 (C-11, C-13), 24.8 (C-32), 26.1 (C-31), 26.2 (C-30), 34.6 (C-33), 38.1 (C-35), 44.2 (C-10, C-12), 45.5 (C-21), 51.3 (C-29), 76.7 (C-16), 96.6 (C-8), 101.6 (C-3), 108.6 (C-4a), 111.4 (C-25, C-27), 111.5 (C-6), 112.9 (C-9), 118.8 (C-15), 119.1 (C-17, C-20), 122.6 (C-23), 126.7 (C-5), 130.9 (C-24, C-28), 140.6 (C-22), 142.8 (C-18, C-19), 148.0 (C-4), 148.5 (C-14), 150.8 (C-26), 151.7 (C-7), 155.0 (C-8a), 165.3 (C-2), and 175.0 (C-34). The FTMS(+) calc. values for C_36_H_41_O_3_N_4_ [M-(I)^-^)]^+^ were 577.3173 and 577.3161. The UV λ^max^ (nm, CH_3_CN) value was 634. The ε (cm^−1^ M^−1^) value was 113,500. Φ_F_ = 0.05.

#### 3.2.12. Synthesis of 4-(Cyano((*E*)-7-(diethylamino)-4-((*E*)-4-((6-((2,5-dioxopyrrolidin-1-yl)oxy)-6-oxohexyl)oxy)styryl)-2H-chromen-2-ylidene)methyl)-1-methylpyridin-1-ium Iodide (**14**)

A mixture of compound **12** (160 mg, 0.234 mmol, 1.0 eq), DCC (57 mg, 0.278 mmol, 1.2 eq), and DMAP (3 mg, 0.0231 mmol, 10%) in dry DMF (2 mL) and dry acetonitrile (8 mL) was stirred at room temperature for 5 min. After this reaction time, *N*-hydroxysuccinimide (32 mg, 0.278 mmol, 1.2 eq) was added, and the reaction was stirred at room temperature for 6 h and 30 min. The reaction was monitored by TLC, using CH_2_Cl_2_/CH_3_OH (9:1) as the eluent. The reaction mixture was evaporated to dryness, and the residue was purified by flash chromatography using CH_2_Cl_2_/CH_3_OH (95:5) and CH_2_Cl_2_/CH_3_OH (9:1) as eluents, to yield 4-(cyano((*E*)-7-(diethylamino)-4-((*E*)-4-((6-((2,5-dioxopyrrolidin-1-yl)oxy)-6-oxohexyl)oxy)styryl)-2*H*-chromen-2-ylidene)methyl)-1-methylpyridin-1-ium iodide, as a dark green solid (165 mg, 91%).

The ^1^H NMR (400 MHz, CDCl_3_, δ, ppm) values were as follows: 1.25 (6H, t, *J* = 6.9, N(CH_2_*CH*_3_)_2_), 1.54 (2H, m, H-31), 1.83 (4H, m, H-30, H-32), 2.67 (2H, t, *J* = 7.2, H-33), 2.86 (4H, s, H-36, H-37), 3.59 (4H, q, *J* = 6.9, N(*CH*_2_CH_3_)_2_), 4.02 (2H, t, *J* = 6.1, H-29), 4.13 (3H, s, H-21), 6.77 (1H, dd, *J* _6,5_ = 9.2, H-6), 6.90 (2H, d, *J* _25,24_ = 8.5, H-25, H-27), 6.97 (1H, sl, H-8), 7.03 (1H, s, H-3), 7.26 (1H, d, *J*_9,22_ = 15.8, H-9), 7.38 (1H, d, *J*_22,9_ = 15.8, H-22), 7.56 (2H, d, *J*_24,25_ = 8.5, H-24, H-28), 7.81 (1H, *J*_5,6_ = 9.2, H-5), 7.98 (2H, d, *J*_17,18_ = 6.2, H-17, H-20), and 8.57 (1H, d, *J*_18,17_ = 6.3, H-18, H-19). The ^13^C NMR (100 MHz, CDCl_3_, δ ppm) values were as follows: 13.2 (C-11, C-13), 24.7 (C-32), 25.6 (C-31), 26.0 (C-36, C-37), 29.0 (C-30), 31.2 (C-33), 46.0 (C-10, C-12), 46.7 (C-21), 68.1 (C-29), 79.1 (C-16), 98.2 (C-8), 104.2 (C-3), 109.5 (C-4a), 112.5 (C-6), 115.3 (C-25, C-27), 117.0 (C-9), 119.2 (C-15), 120.6 (C-17, C-20), 126.6 (C-5), 128.3 (C-23), 130.4 (C-24, C-28), 139.7 (C-22), 143.1 (C-18, C-19), 148.5 (C-4), 149.7 (C-14), 152.9 (C-7), 156.1 (C-8a), 161.3 (C-26), 167.0 (C-2), 168.8 (C-34), and 169.6 (C-35, C-38). The FTMS(+) calc. values for C_39_H_41_O_6_N_4_ [M + H]^+^ were 661.3021 and 661.3013. The UV λ^max^ (nm, CH_3_CN) values were as follows: 589 and 615. The ε (cm^−1^ M^−1^) value was 55,000. Φ_F_ = 0.10.

#### 3.2.13. Synthesis of 4-(Cyano((*E*)-7-(diethylamino)-4-((*E*)-4-((6-((2,5-dioxopyrrolidin-1-yl)oxy)-6-oxohexyl)(methyl)amino)styryl)-2H-chromen-2-ylidene)methyl)-1-methyl-pyridin-1-ium Iodide (**15**)

A mixture of compound **13** (48 mg, 0.0681 mmol, 1.0 eq), DCC (17 mg, 0.0817 mmol, 1.2 eq), and DMAP (0.08 mg, 6.81 × 10^−4^ mmol, 10%) in dry DMF (2 mL) and dry acetonitrile (8 mL) was stirred at room temperature for 5 min. After this reaction time, *N*-hydroxysuccinimide (9.4 mg, 0.0817 mmol, 1.2 eq) was added, and the reaction was stirred at room temperature for 23 h. The reaction was monitored by TLC, using CHCl_3_/CH_3_OH (9:1) as the eluent. The reaction mixture was evaporated to dryness, and the residue was purified by flash chromatography using CHCl_3_/CH_3_OH (9:1) and CHCl_3_/CH_3_OH (8:2) as eluents, to yield 4-(cyano((*E*)-7-(diethylamino)-4-((*E*)-4-((6-((2,5-dioxopyrrolidin-1-yl)oxy)-6-oxohexyl)(methyl)amino)styryl)-2*H*-chromen-2-ylidene)methyl)-1-methylpyridin-1-ium iodide, as a dark blue solid (53 mg, 97%).

The ^1^H NMR (400 MHz, CDCl_3_, δ, ppm) values were as follows: 1.25 (6H, t, *J* = 7.1, N(CH_2_*CH*_3_)_2_), 1.46–1.52 (2H, m, H-31), 1.62–1.72 (2H, m, H-32), 1.77–1.85 (2H, m, H-30) 2.64 (2H, t, *J* = 7.2, H-33), 2.86 (4H, s, H-37, H-38), 3.04 (3H, s, H-35), 3.42 (2H, t, *J* = 7.2, H-29), 3.57 (4H, q, *J* = 7.1, N(*CH*_2_CH_3_)_2_), 4.11 (3H, s, H-21), 6.66 (2H, d, *J*_25,24_ = 8.9, H-25, H-27), 6.77 (1H, d, *J*_6,5_ = 9.3, *J*_6,8_ = 2.3, H-6) 6.90 (1H, d, *J*_8,6_ = 2.3 H-8), 7.03 (1H, s, H-3), 7.15 (1H, d, *J*_9,22_ = 15.7, H-9), 7.41 (1H, d, *J*_22,9_ = 15.7, H-22), 7.51 (2H, *J*_24,25_ = 8.9, H-24, H-28), 7.83 (1H, d, *J*_5,6_ = 9.3, H-5), 7.93 (2H, d, *J*_17,18_ = 5.2, H-17, H-20), and 8.45 (2H, d, *J*_18,17_ = 5.1, H-18, H-19). The ^13^C NMR(100 MHz, CDCl_3_, δ ppm) values were as follows: 13.0 (C-11, C-13), 24.6 (C-32), 25.8 (C-37, C-38), 26.2 (C-31), 26.7 (C-30), 29.8 (C-33), 38.7 (C-35), 45.6 (C-10, C-12), 46.3 (C-21), 52.3 (C-29), 77.4 (C-16), 97.9 (C-8), 103.0 (C-3), 109.4 (C-4a), 112.0 (C-25, C-27), 112.2 (C-6), 113.2 (C-9), 119.3 (C-15), 120.0 (C-17, C-20), 123.2 (C-23), 126.3 (C-5), 130.8 (C-24, C-28), 141.0 (C-22), 142.4 (C-18, C-19), 148.9 (C-4), 149.8 (C-14), 151.1 (C-26), 152.5 (C-7), 156.0 (C-8a), 166.3 (C-2), 168.6 (C-34), and 169.4 (C-36, C-39). The FTMS(+) calc. values for C_40_H_44_O_5_N_5_ [M-(I^-^)]^+^ were 674.3337 and 674.3331. The UV λ^max^ (nm, CH_3_CN) value was 634. The ε (cm^−1^ M^−1^) value was 113,500. Φ_F_ = 0.07.

#### 3.2.14. Synthesis of 1-(7-(Diethylamino)-4-methyl-2H-chromen-2-ylidene)-piperidin-1-ium Nitrate (**16**)

The synthesis of 1-(7-(diethylamino)-4-methyl-2*H*-chromen-2-ylidene)-piperidin-1-ium nitrate (**16**) was performed according to the method described by Eustáquio et al. [28].

#### 3.2.15. Synthesis of (*E*)-1-(4-(4-((5-Carboxypentyl)oxy)styryl)-7-(diethylamino)-2H-chromen-2-ylidene)piperidin-1-ium Nitrate (**17**)

The synthesis of (*E*)-1-(4-(4-((5-carboxypentyl)oxy)styryl)-7-(diethylamino)-2*H*-chromen-2-ylidene)piperidin-1-ium nitrate (**17**) was performed according to the method described by Eustáquio et al. [28].

#### 3.2.16. Synthesis of (*E*)-1-(4-(4-((5-Carboxypentyl)(methyl)amino)styryl)-7-(diethylamino)-2H-chromen-2-ylidene)piperidin-1-ium Nitrate (**18**)

A mixture of compound **16** (85 mg, 0.235 mmol, 1.0 eq), 6-((4-formylphenyl)(methyl)amino)hexanoic acid **5** (58 mg, 0.235 mmol, 1.0 eq), and piperidine (23 µL, 0.235 mmol, 1.0 eq) in dry acetonitrile (10 mL) was stirred at 85 °C, for a period of 24 h. The reaction mixture, at room temperature, was then acidified with 6 M HCl and was continuously monitored by TLC, using CHCl_3_/CH_3_OH/H_2_O (65:10:1) as the eluent. The reaction mixture was evaporated to dryness, and the residue was purified by flash chromatography, using CHCl_3_/CH_3_OH (9:1), CHCl_3_/CH_3_OH (8:2), CHCl_3_/CH_3_OH/H_2_O (65:10:1), CHCl_3_/CH_3_OH/H_2_O (65:20:2), and CHCl_3_/CH_3_OH/H_2_O (65:35:5) as eluents, to yield (*E*)-1-(4-(4-((5-carboxypentyl)(methyl)amino)styryl)-7-(diethylamino)-2*H*-chromen-2-ylidene)piperidin-1-ium nitrate, as an orange solid (122 mg, 88%).

The ^1^H NMR (400 MHz, CD_3_OD, δ, ppm) values were as follows: 1.26 (6H, t, *J* = 7.2, N(CH_2_*CH*_3_)_2_), 1.36–1.43 (2H, m, H-28), 1.62–170 (4H, m, H-27, H-29), 1.83 (6H, m, H-15, H-16, H-17), 2.25 (2H, t, *J*_30,29_ = 7.4, H-30), 3.05 (1H, s, H-32), 3.46 (2H, t, *J*_26,27_ = 7.4, H-26), 3.57 (4H, q, *J* = 7.2, N(*CH*_2_CH_3_)_2_), 3.95 (4H, m, H-14, H-18), 6.76 (2H, d, *J*_22,21_ = 9.0, H-22, H-24), 6.84 (1H, d, *J*_8,6_ = 2.6, H-8), 6.95 (1H, s, H-3), 6.99 (1H, dd, *J*_6,5_ = 9.4, *J*_6,8_ = 2.6, H-6), 7.42 (1H, d, *J*_9,19_ = 15.7, H-9), 7.66 (2H, d, *J*_21,22_ = 9.0, H-21, H-25), 7.87 (1H, d, *J*_19,9_ = 15.7, H-19), and 8.07 (1H, d, *J*_5,6_ = 9.4, H-5). The ^13^C NMR (100 MHz, CD_3_OD, δ, ppm) values were as follows: 12.7 (C-11, C-13), 24.8 (C-16), 26.7 (C-29), 27.0 (C-15, C-17), 27.7 (C-27), 27.8 (C-28), 36.8 (C-30), 38.7 (C-32), 46.0 (C-10, C-12),48.1 (C-14/C-18), 53.1 (C-26), 91.1 (C-3), 98.0 (C-8), 109.1 (C-4a), 112.8 (C-6), 113.0 (C-22, C-24), 113.4 (C-9), 124.4 (C-20), 127.8 (C-5), 131.8 (C-21, C-25), 144.2 (C-19), 152.8 (C-23), 154.0 (C-7), 155.1 (C-4), 156.3 (C-8a), 161.3 (C-2), and 178.0 (C-31). The FTMS(+) calc. values for C_33_H_44_O_3_N_3_ [M-(NO_3_^-^)]^+^ were 530.3377 and 530.3369. The UV λ^max^ (nm, CH_3_CN) value was 489. The ε (cm^−1^ M^−1^) value was 64,000. Φ_F_ = 0.54.

#### 3.2.17. Synthesis of (*E*)-1-(7-(Diethylamino)-4-(4-((6-((2,5-dioxopyrrolidin-1-yl)oxy)-6-oxohexyl)oxy)styryl)-2H-chromen-2-ylidene)piperidin-1-ium Nitrate (**19**)

The synthesis of (*E*)-1-(7-(diethylamino)-4-(4-((6-((2,5-dioxopyrrolidin-1-yl)oxy)-6-oxohexyl)oxy)styryl)-2*H*-chromen-2-ylidene)piperidin-1-ium nitrate (**19**) was performed according to the method described by Eustáquio et al. [28].

#### 3.2.18. Synthesis of (*E*)-1-(7-(Diethylamino)-4-(4-((6-((2,5-dioxopyrrolidin-1-yl)oxy)-6-oxohexyl)(methyl)amino)styryl)-2H-chromen-2-ylidene)piperidin-1-ium Nitrate (**20**)

A mixture of compound **18** (65 mg, 0.109 mmol, 1.0 eq), DCC (27 mg, 0.132 mmol, 1.2 eq), and DMAP (0.13 mg, 1.09 × 10^−3^ mmol, 10%) in dry DMF (2 mL) and dry acetonitrile (8 mL) was stirred at room temperature for 5 min. After this reaction time, *N*-hydroxysuccinimide (15 mg, 0.132 mmol, 1.2 eq) was added, and the reaction was stirred at room temperature for 24 h. The reaction was monitored by TLC, using CH_2_Cl_2_/CH_3_OH (9:1) as the eluent. The reaction mixture was evaporated to dryness, and the residue was purified by flash chromatography using CH_2_Cl_2_/CH_3_OH (9:1) and CH_2_Cl_2_/CH_3_OH (8:2) as eluents, to yield ((*E*)-1-(7-(diethylamino)-4-(4-((6-((2,5-dioxopyrrolidin-1-yl)oxy)-6-oxohexyl)(methyl)amino)styryl)-2*H*-chromen-2-ylidene)piperidin-1-ium nitrate, as an orange solid (68 mg, 97%).

The ^1^H NMR (400 MHz, CDCl_3_, δ, ppm) values were as follows: 1.26 (6H, t, *J* = 7.1, N(CH_2_*CH*_3_)_2_), 1.45–1.50 (2H, m, H-28), 1.61–1.69 (2H, m, H-29), 1.76–180 (8H, m, H-27, H-15, H-16, H-17), 2.63 (2H, t, *J*_30,29_ = 7.2, H-30), 2.86 (4H, s, H-34, H-35), 3.02 (1H, s, H-32), 3.41 (2H, t, *J*_26,27_ = 7.3, H-26), 3.49 (4H, q, *J* = 7.1, N(*CH*_2_CH_3_)_2_), 3.97 (4H, m, H-14, H-18), 6.61 (1H, d, *J*_8,6_ = 2.5, H-8), 6.68 (2H, d, *J*_22,21_ = 9.0, H-22, H-24), 6.80 (1H, dd, *J*_6,5_ = 9.4, *J*_6,8_ = 2.6, H-6), 7.02 (1H, s, H-3), 7.18 (1H, d, *J*_9,19_ = 15.7, H-9), 7.66 (2H, d, *J*_21,22_ = 9.0, H-21, H-25), 7.84 (1H, d, *J*_5,6_ = 9.4, H-5), and 7.97 (1H, d, *J*_19,9_ = 15.7, H-19). The ^13^C NMR (100 MHz, CDCl_3_, δ, ppm) values were as follows: 12.7 (C-11, C-13), 23.8 (C-16), 24.6 (C-29), 25.8 (C-34, C-35), 26.1 (C-15, C-17), 26.2 (C-28), 26.6 (C-27), 31.0 (C-30), 38.7 (C-32), 45.2 (C-10, C-12), 47.7 (C-14, C-18), 52.3 (C-26), 91.2 (C-3), 97.1 (C-8), 108.2 (C-4a), 111.5 (C-6), 112.0 (C-22, C-24), 112.1 (C-9), 123.7 (C-20), 126.5 (C-5), 131.1 (C-21, C-25), 144.0 (C-19), 151.2 (C-23), 152.3 (C-7), 153.5 (C-4), 154.7 (C-8a), 159.8 (C-2), 168.6 (C-31), and 169.4 (C-33, C-36). The FTMS(+) calc. values for C_37_H_47_O_5_N_4_ [M-(NO_3_^-^)]^+^ were 627.3541 and 627.3531. The UV λ^max^ (nm, CH_3_CN) value was 487. The ε (cm^−1^ M^−1^) value was 640,00. Φ_F_ = 0.56.

### 3.3. Quantum Chemical Calculations

To shed light into the observed photophysical properties, density functional theory (DFT) and time-dependent density functional theory (TD-DFT) and calculations were performed by the Gaussian 16 package [43] with the hybrid PBE0 functional [44] and the standard 6-31G(d,p) basis set being used for geometry optimizations of both the ground and first excited states. The solvent effects were considered by the implicit polarized continuum model (PCM) [45,46]. Following geometry optimizations, the vibration analysis was conducted presenting no imaginary frequencies, thus attesting the structures as true minima. For the TD-DFT spectra calculations, the larger 6–311+G(d,p) basis set was used.

## 4. Conclusions

In summary, our aim to extend the delocalization the π-electron system led us to create and produce novel derivatives of 4-styrylcoumarin. These derivatives exhibited absorption and emission characteristics at extended wavelengths and featured significant Stokes shifts. All of this was accomplished through a straightforward, efficient, and cost-effective synthetic approach. The results derived from the UV/Vis spectra indicated that the intermediates 2-(7-(diethylamino)-4-methyl-2*H*-chromen-2-ylidene)malononitrile (**3**), (*E*)-4-(cyano(7-(diethylamino)-4-methyl-2*H*-chromen-2-ylidene)methyl)-1-methylpyridin-1-ium iodide (**11**), and 1-(7-(diethylamino)-4-methyl-2*H*-chromen-2-ylidene)piperidin-1-ium nitrate (**16**) hold significant promise as cost-effective and innovative intermediate compounds in the synthesis of novel fluorescent labels for biomolecules. The utilization of DFT and TDDFT calculations provided valuable insights into the observed photophysical properties, specifically the large Stokes shifts. These shifts were attributed to substantial structural relaxation observed in the excited states of the molecules, offering a rationalization for the phenomenon.

We are currently working on synthesizing other red-shifted coumarin fluorescent labels for biomolecules, aiming to enhance their characteristics. We anticipate sharing a concise report on the outcomes in the near future.

## Figures and Tables

**Table 1:** Spectroscopic properties of 7-diethylamino-4-methylcoumarin derivatives.

Compound	λ_abs_ ^a^ (nm)	λ_em_ ^b^ (nm)	Stokes Shift (nm, cm^−1^)	ε ^c^ (cm^−1^ M^−1^)	Φ_F_ ^d^
**1**	371	434	63, 3913	22,910	0.73
**3**	477	519	42, 1697	38,000	0.05
**6**	519	596	77, 2489	24,000	0.28
**7**	500 (578) ^e^	628	128, 4076	59,000	0.27
**8**	523	597	74, 2370	24,000	0.29
**9**	498 (576) ^e^	624	126, 4055	59,000	0.27
**11**	550	603	53, 1598	80,000	0.36
**12**	593 (615) ^e^	663	70, 1780	55,000	0.07
**13**	634	685	51, 1174	113,500	0.05
**14**	589 (615) ^e^	663	74, 1895	55,000	0.10
**15**	634	683	49, 1132	113,500	0.07
**16**	437	490	53, 2475	21,000	0.14
**17**	406 (476) ^e^	578	172, 7330	30,000	0.90
**18**	489	628	139, 4526	64,000	0.54
**19**	404 (480) ^e^	577	173, 7421	30,000	0.92
**20**	487	628	141, 4610	64,000	0.56

^a^ Absorption maxima in acetonitrile. ^b^ Emission maxima in acetonitrile. ^c^ Molar extinction coefficient at longest wavelength transition. ^d^ Fluorescence quantum yield in ethanol, determined using 7-diethylamino-4-methylcoumarin (Φ_F_ = 0.73 in ethanol) as a standard. ^e^ Shoulder.

## Data Availability

Computational files are available from the authors.

## Supplementary Materials

The following supporting information can be downloaded at: https://www.mdpi.com/article/10.3390/molecules28196822/s1.

## Sample Availability

Samples of most of the compounds are available from the authors.
