# Supplementary material for: Rational Design of Cost-Effective 4-Styrylcoumarin Fluorescent Derivatives for Biomolecule Labeling
Source: Molecules. 2023 Sep 27;28(19):6822. doi: 10.3390/molecules28196822 (PMC10574246; doi:10.3390/molecules28196822)
Supplement: Supplementary file 1 [file molecules-28-06822-s001.zip › molecules-2620742-supplementary.pdf]

Supplementary materials

Rational design of cost-effective 4-styrylcoumarin fluorescent derivatives for biomolecules labeling

Eustáquio R[a], Ramalho JPP [b,c], Caldeira AT [a,b,d], Pereira A[a,b*]

[a] HERCULES Laboratory & IN2PAST—Associate Laboratory for Research and Innovation in Heritage, Arts, Sustainability and Territory, University of Évora, Largo Marquês de Marialva 8, 7000-809 Évora, Portugal.

[b] Department of Chemistry and Biochemistry, School of Sciences and Technology, University of Évora, Rua Romão Ramalho 59, 7000-671 Évora, Portugal.

[c] Laqv-Requimte, University of Évora, Rua Romão Ramalho 59, 7000-671 Évora, Portugal.

[d] City U Macau Chair in Sustainable Heritage & Sino-Portugal Joint Laboratory of Cultural Heritage Conservation Science, University of Évora, Largo Marquês de Marialva 8, 7000-809 Évora, Portugal

* Corresponding author.

E-mail address: amlp@uevora.pt

1. NMR spectra 2

2. Mass spectra 11

3. Quantum chemical calculations 15

1. NMR spectra

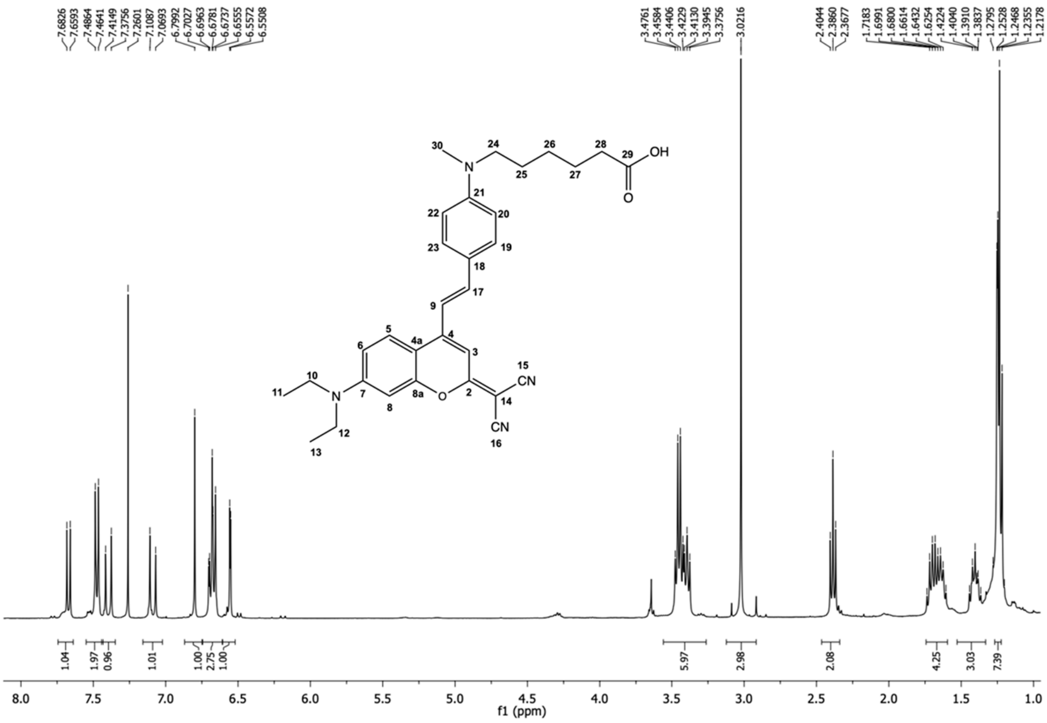

Figure S1 ^{1}H-NMR spectrum of **7** ($CDCl_3$, 400 MHz).

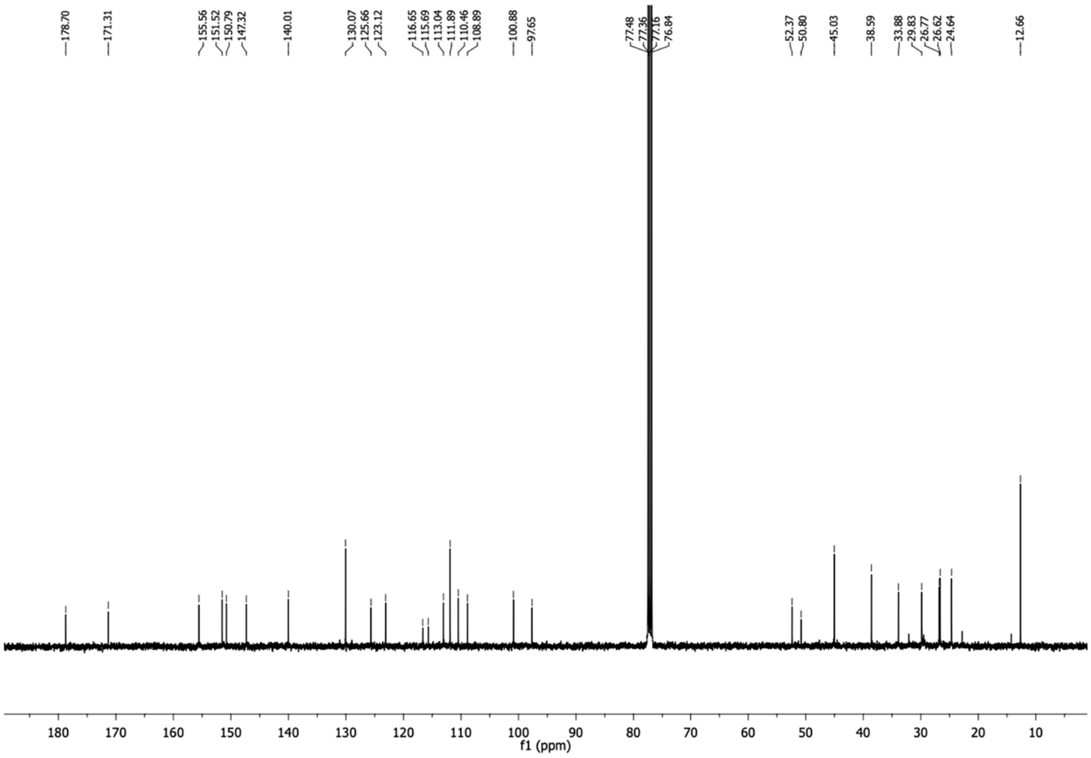

Figure S2 ^{13}C-NMR spectrum of **7** ($CDCl_3$, 100 MHz).

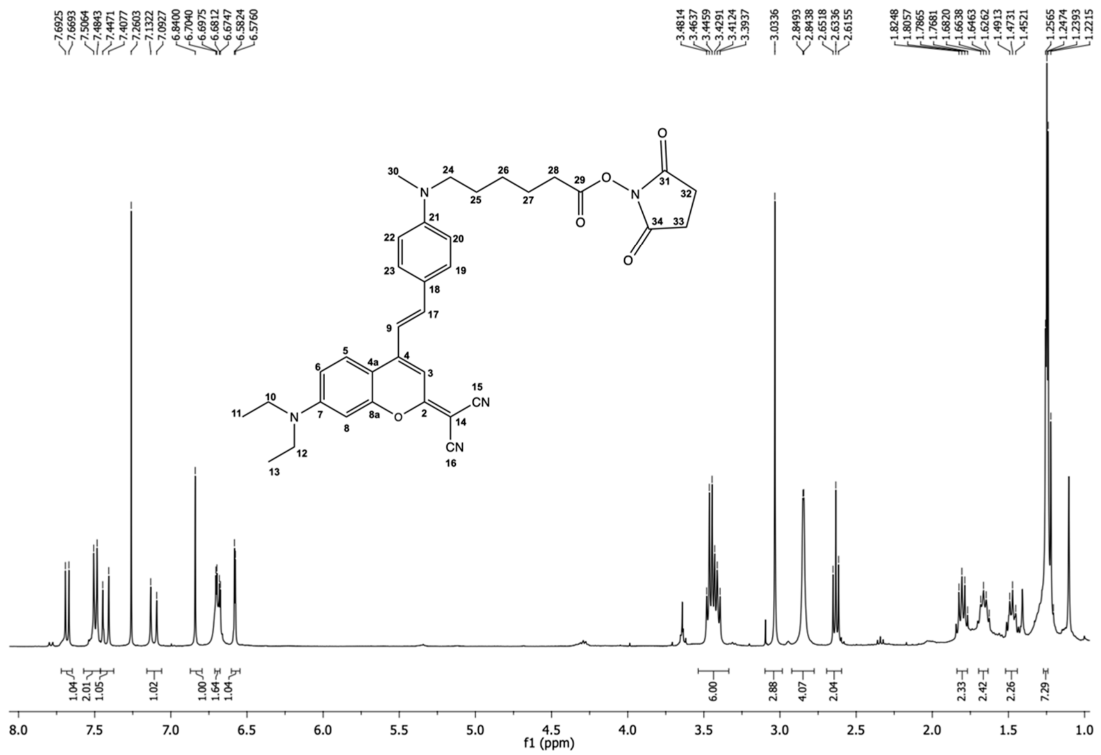

Figure S3 ^{1}H-NMR spectrum of **9** ($CDCl_3$, 400 MHz).

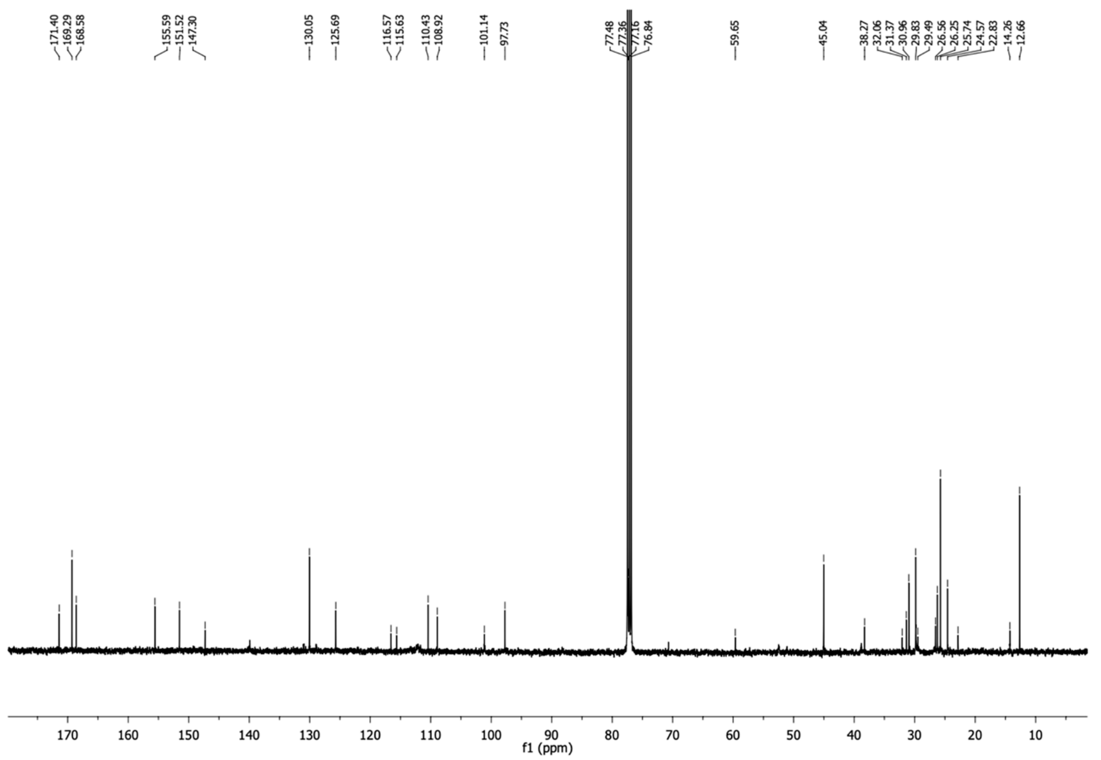

Figure S4 ^{13}C-NMR spectrum of **9** ($CDCl_3$, 100 MHz).

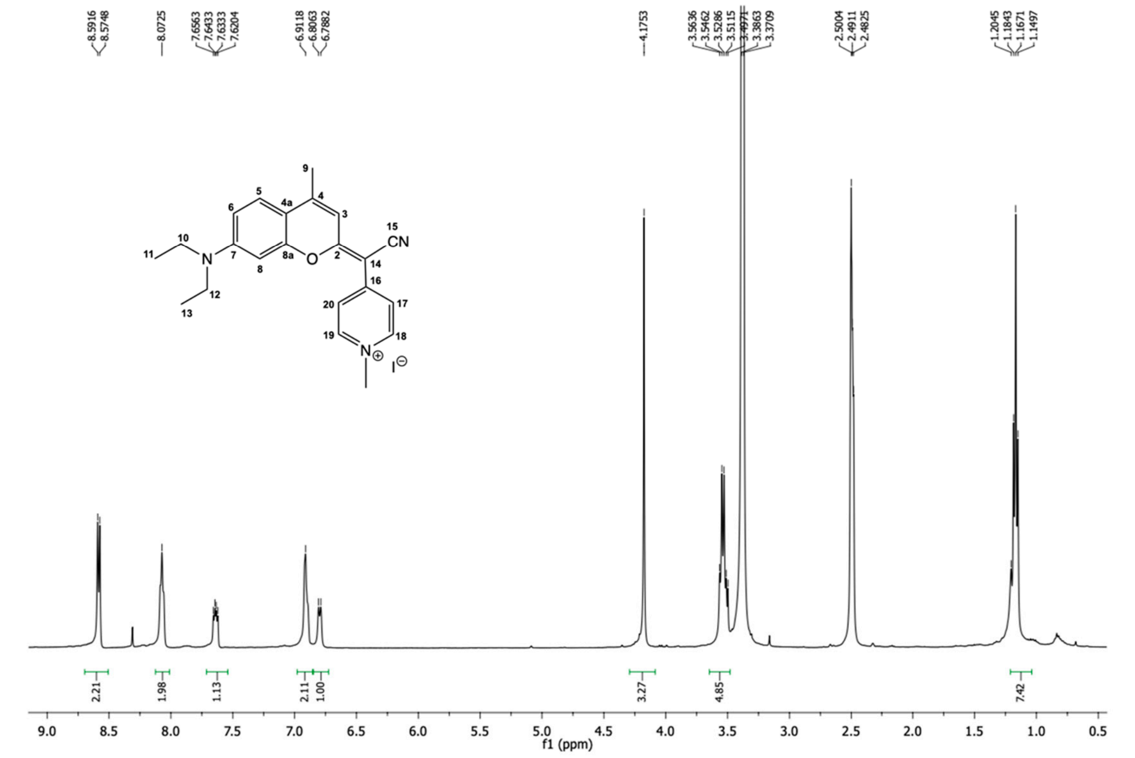

Figure S5 ^{1}H-NMR spectrum of **11** ($(CD_3)_2SO$, 400 MHz).

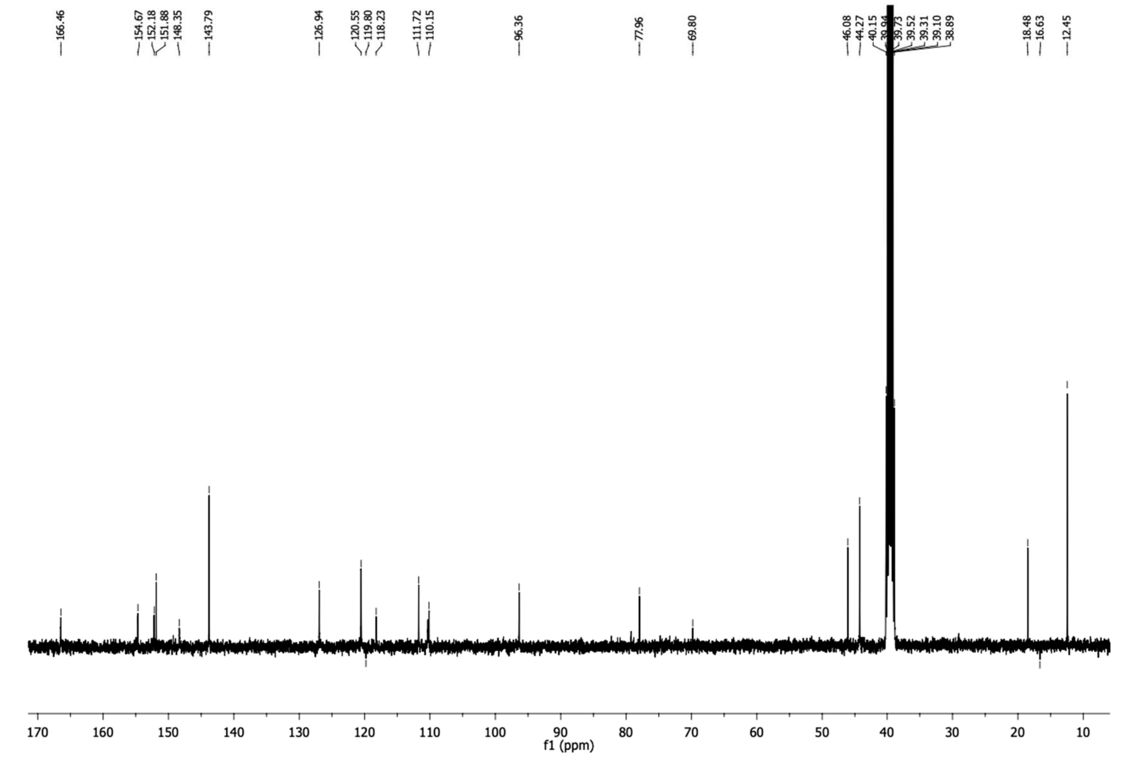

Figure S6 ^{13}C-NMR spectrum of **11** ($(CD_3)_2SO$, 100 MHz).

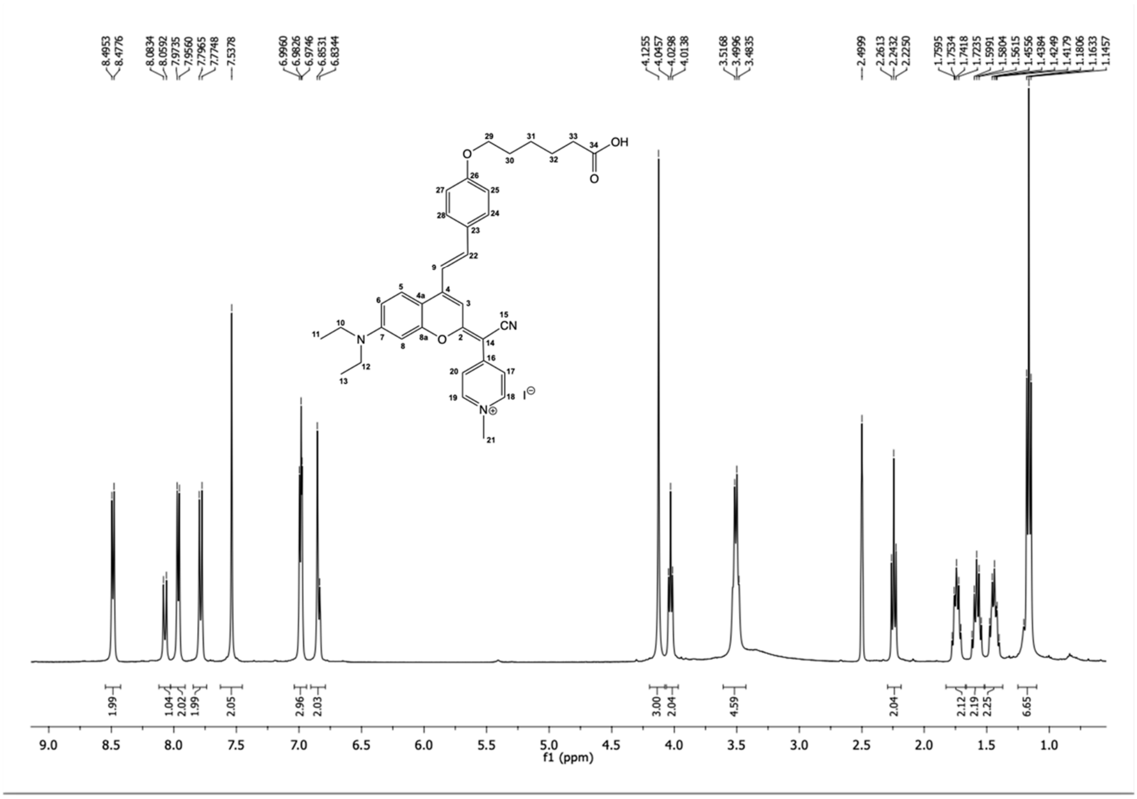

Figure S7 ^{1}H-NMR spectrum of **12** ($(CD_3)_2SO$, 400 MHz).

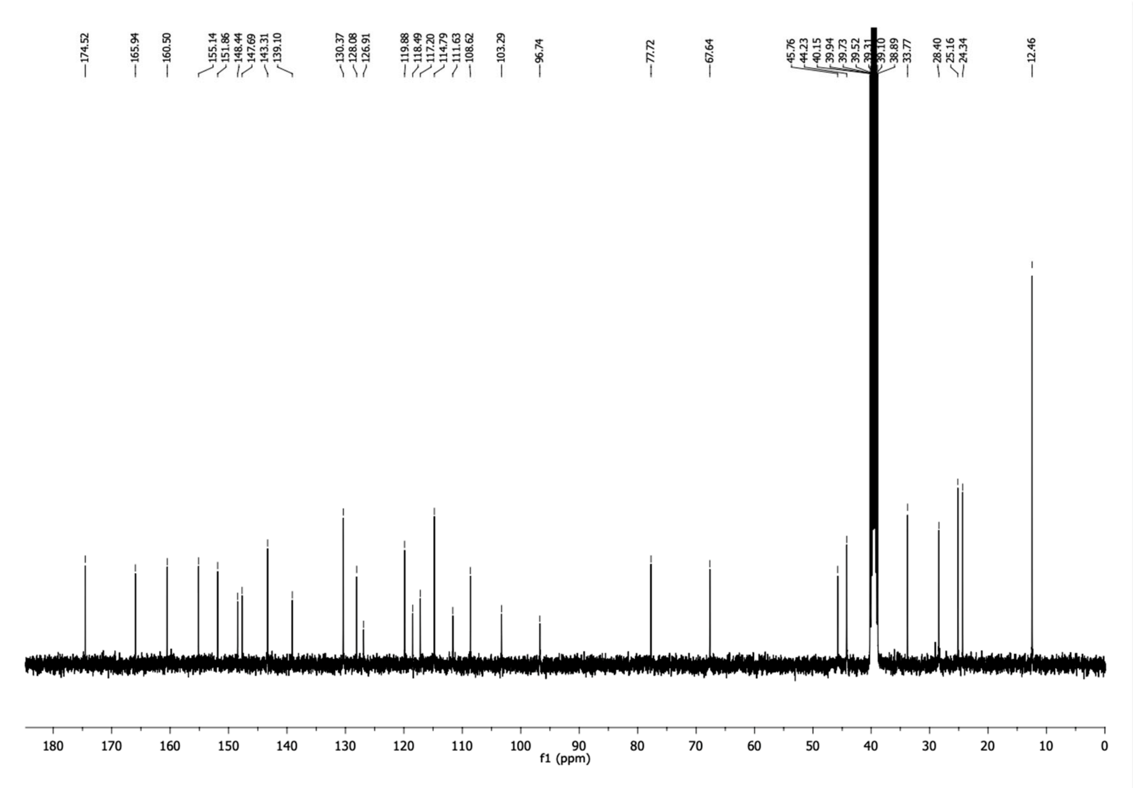

Figure S8 ^{13}C-NMR spectrum of **12** ($(CD_3)_2SO$, 100 MHz).

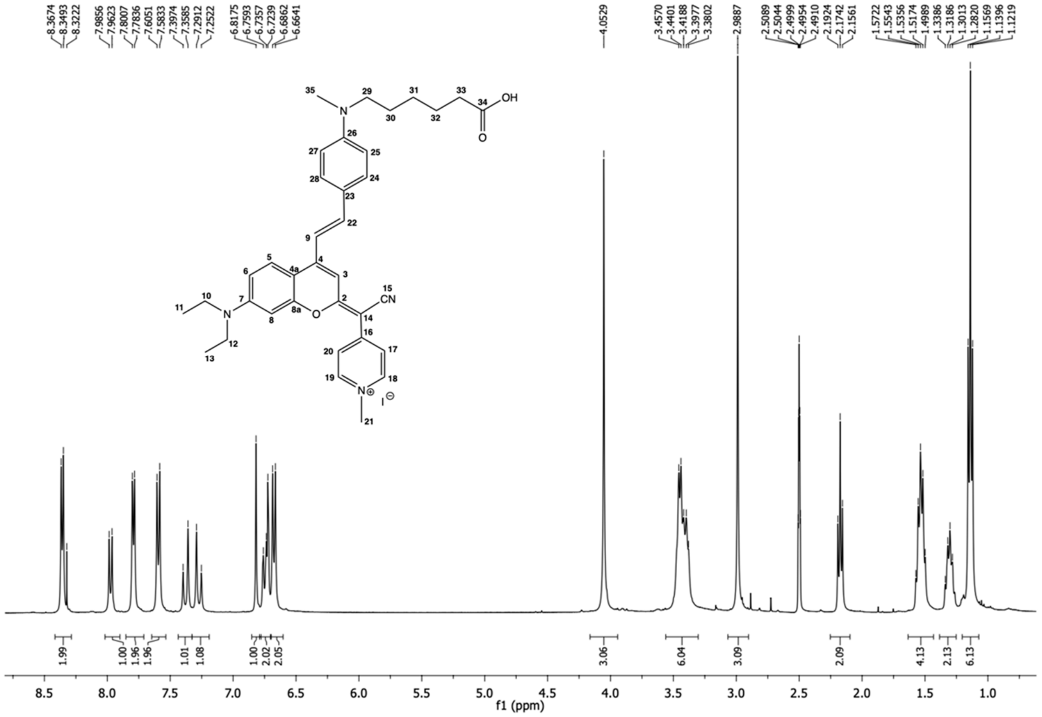

Figure S9 ^{1}H-NMR spectrum of **13** ($(CD_3)_2SO$, 400 MHz).

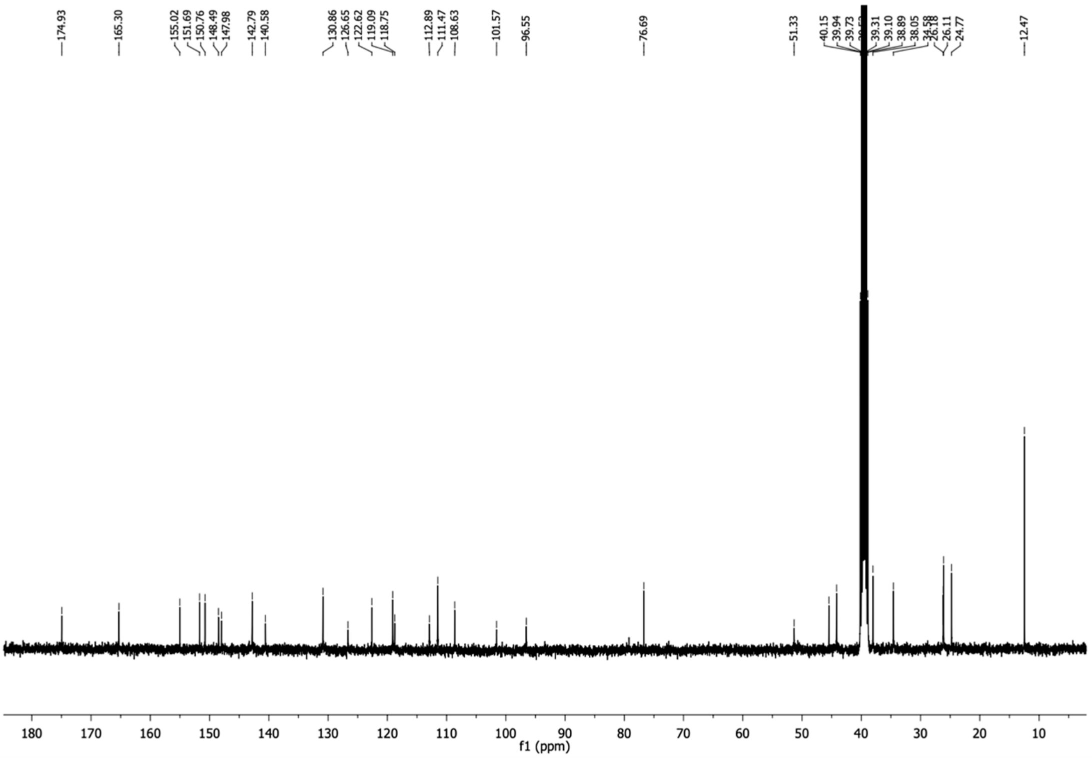

Figure S10 ^{13}C-NMR spectrum of **13** ($(CD_3)_2SO$, 100 MHz).

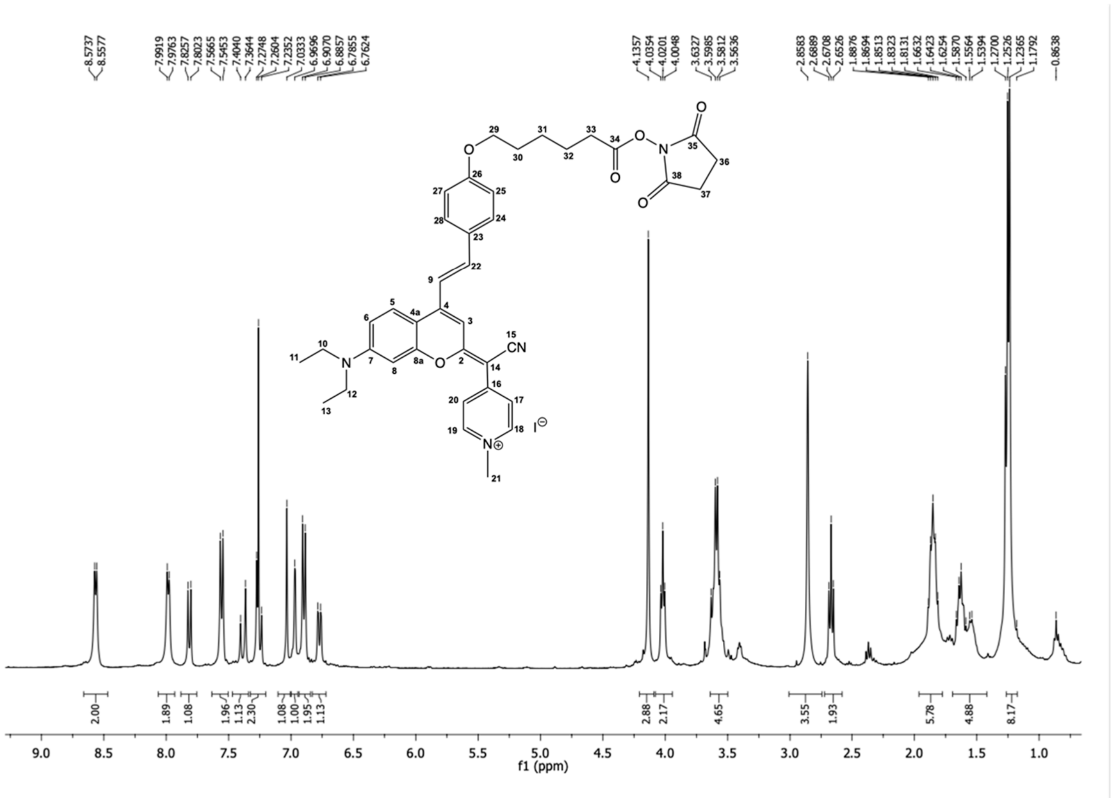

Figure S11 ^{1}H-NMR spectrum of **14** ($CDCl_3$, 400 MHz).

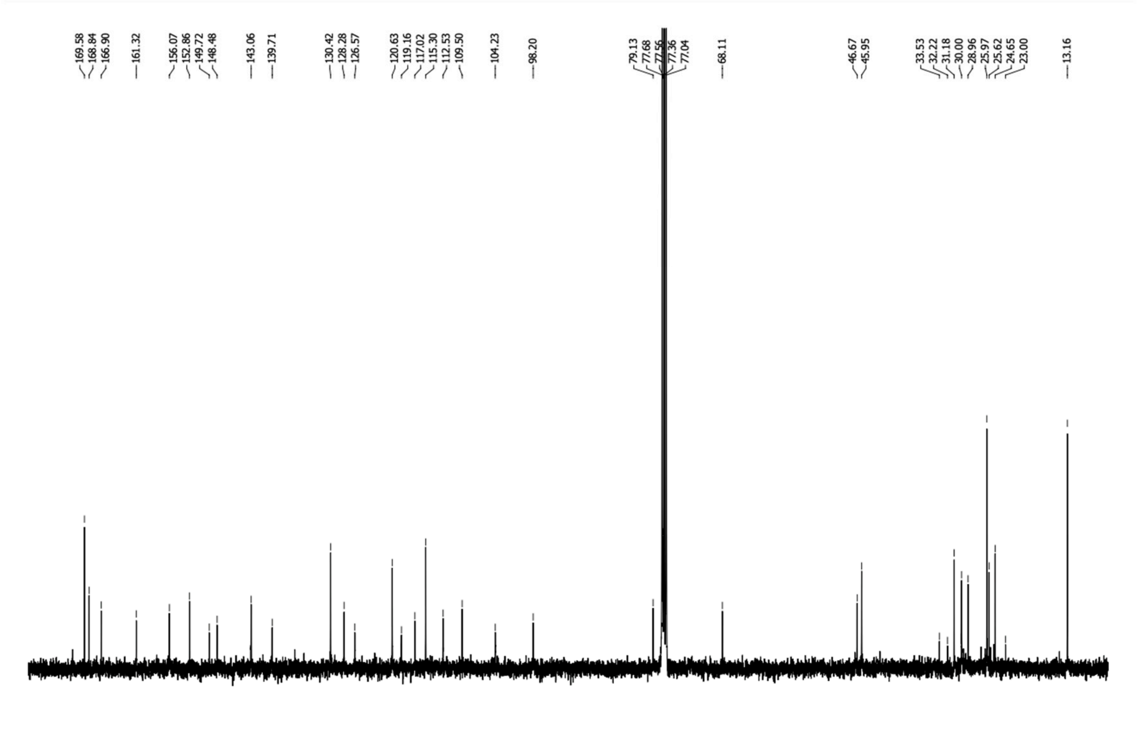

Figure S12 ^{13}C-NMR spectrum of **14** ($CDCl_3$, 100 MHz).

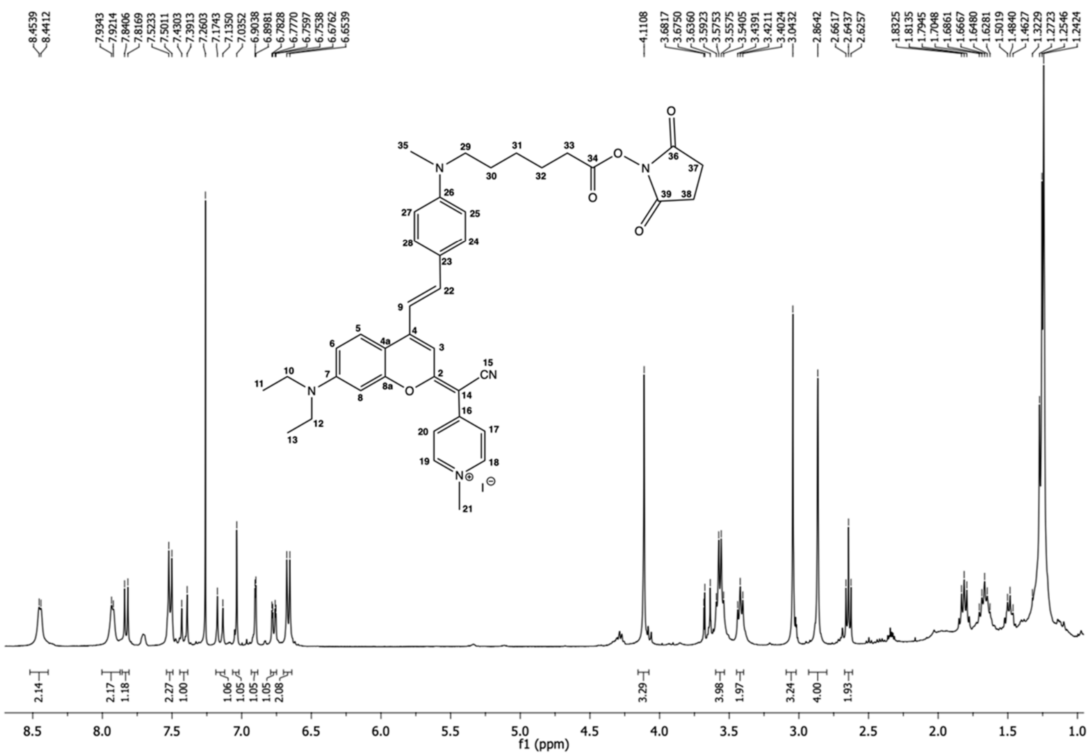

Figure S13 ^{1}H-NMR spectrum of **15** ($CDCl_3$, 400 MHz).

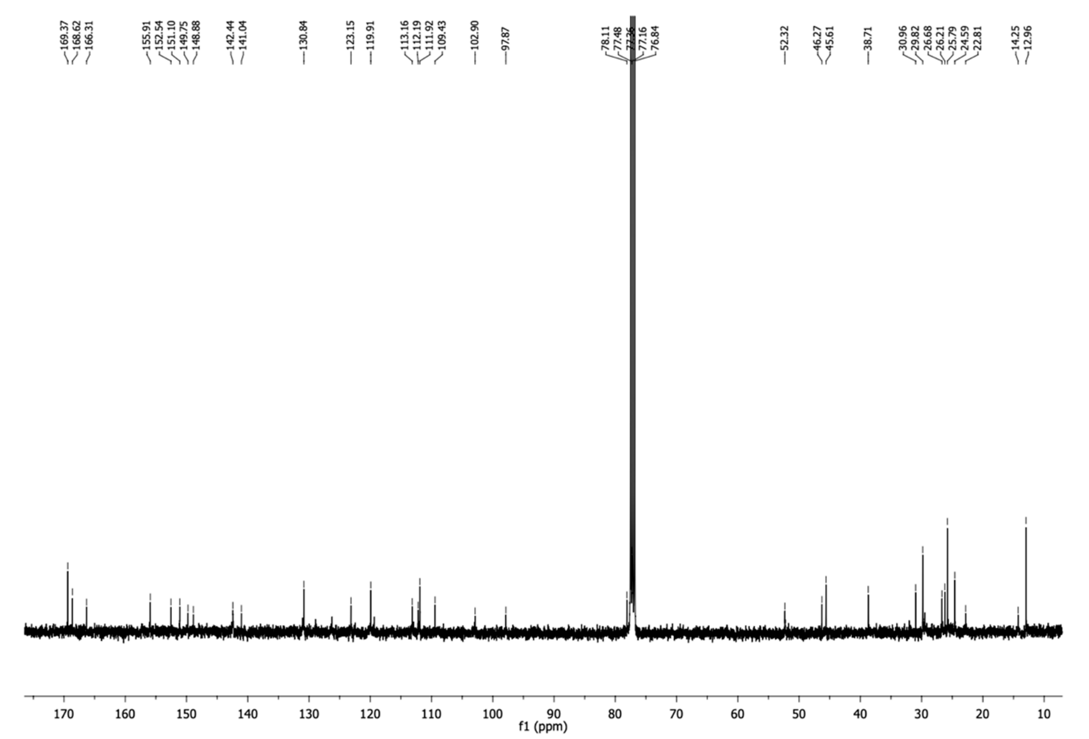

Figure S14 ^{13}C-NMR spectrum of **15** ($CDCl_3$, 100 MHz).

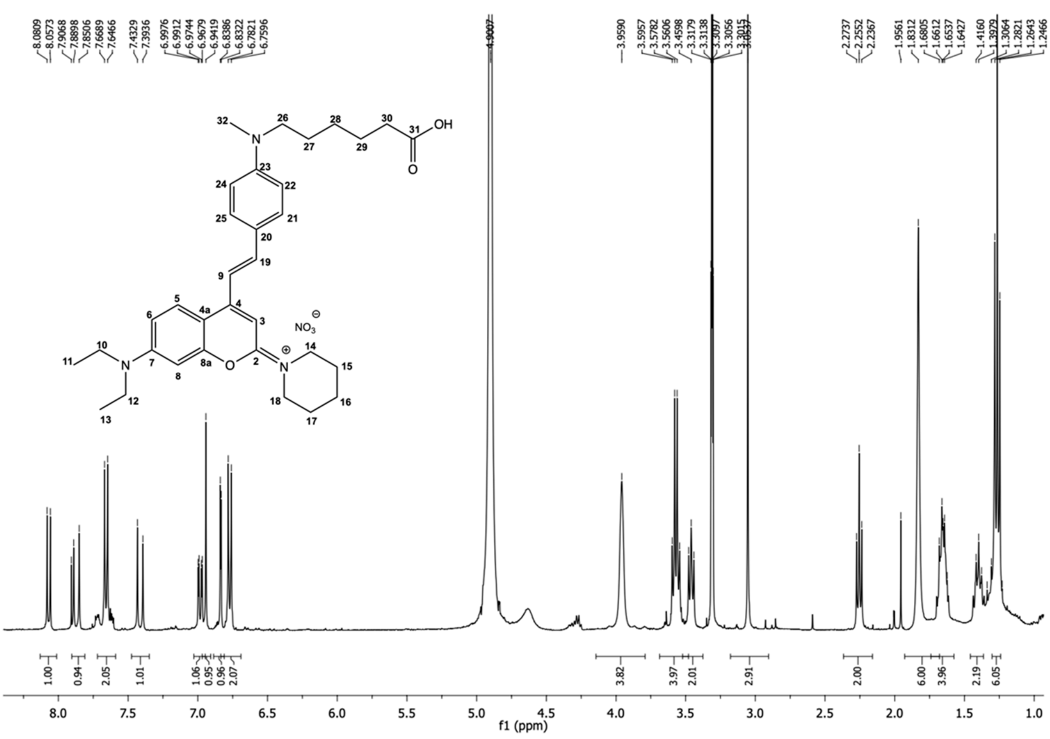

Figure S15 ^{1}H-NMR spectrum of **18** (CD_3OD, 400 MHz).

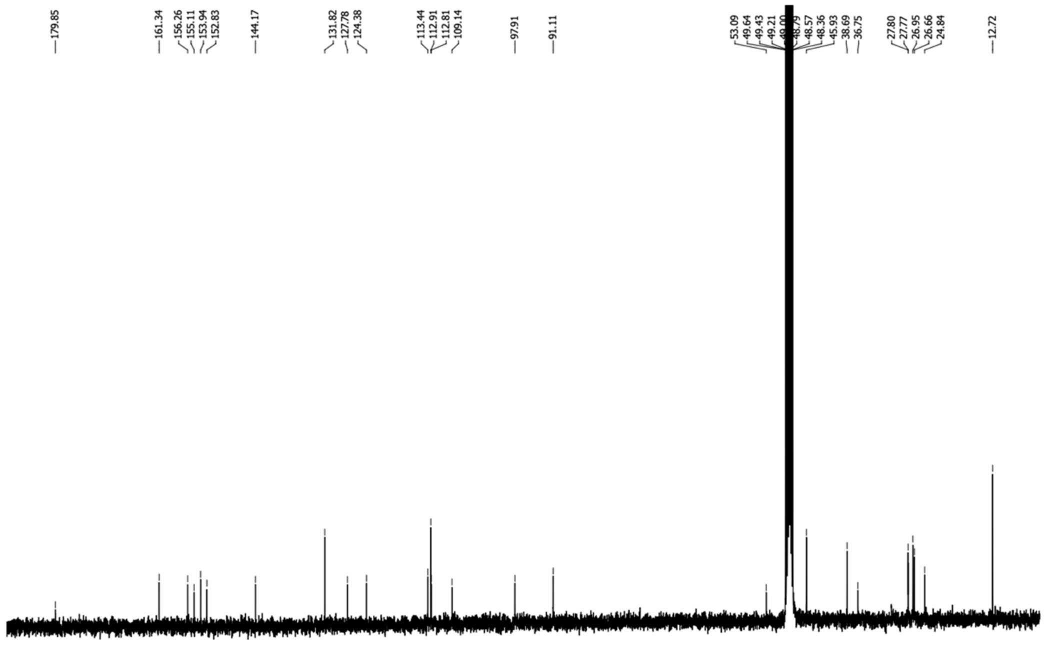

Figure S16 ^{13}C-NMR spectrum of **18** (CD_3OD, 100 MHz).

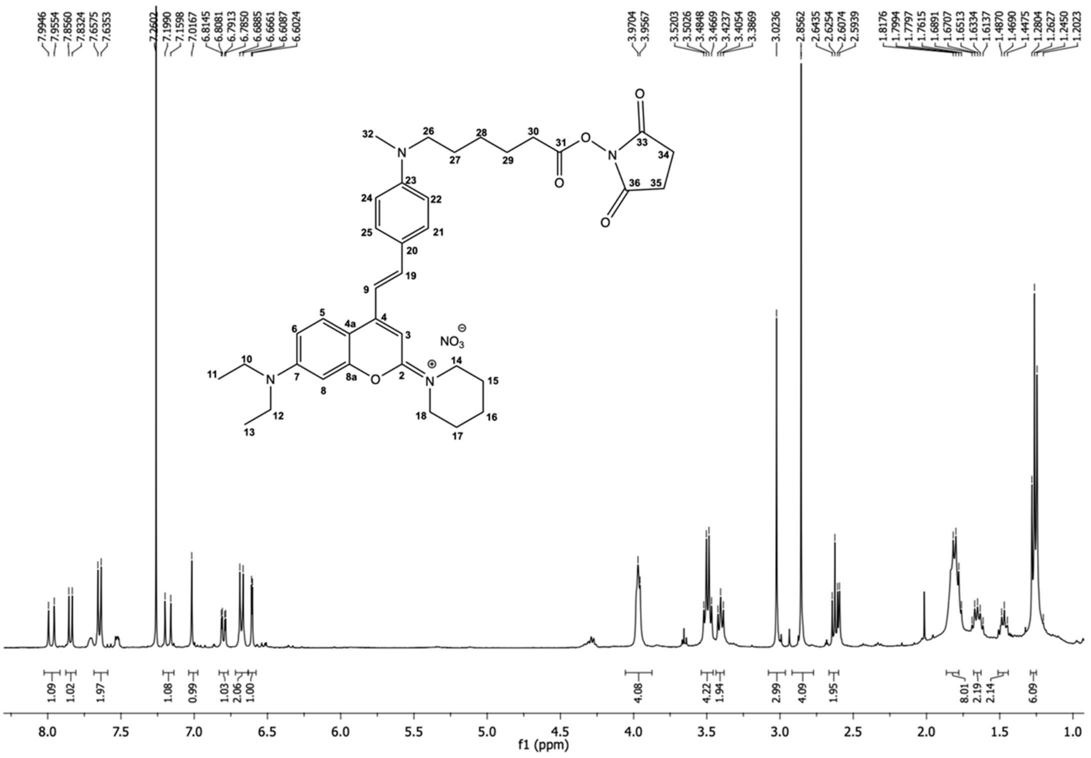

Figure S17 ^{1}H-NMR spectrum of **20** ($CDCl_3$, 400 MHz).

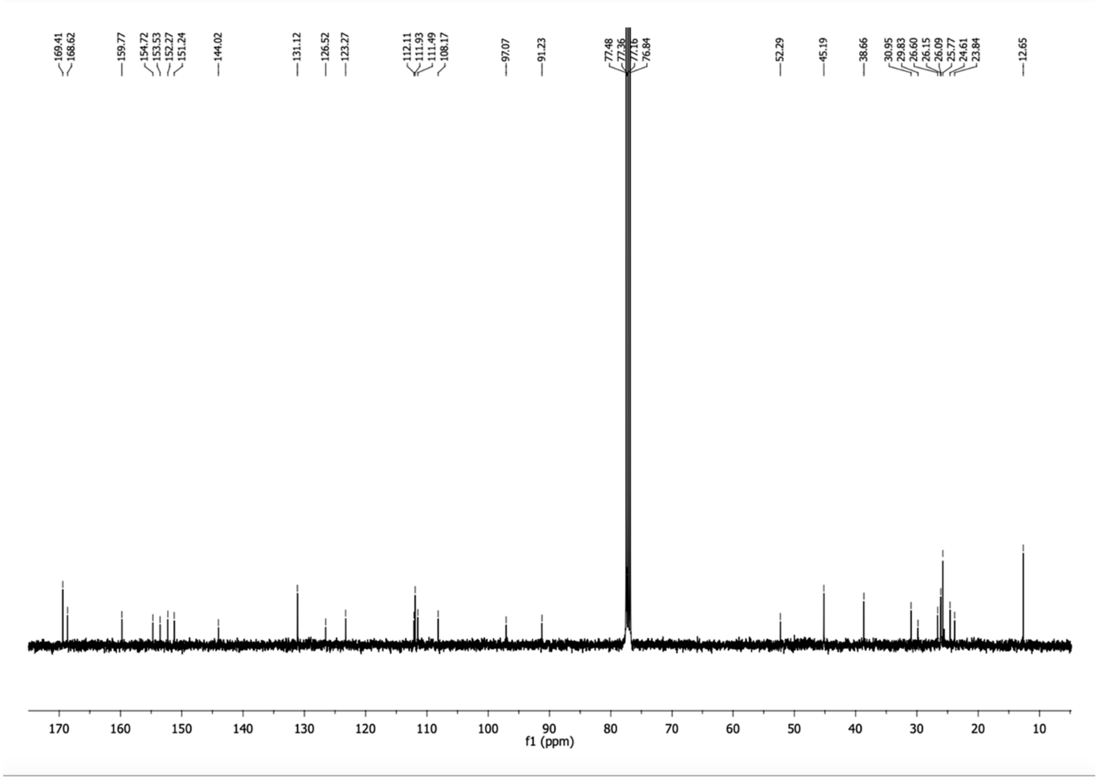

Figure S18 ^{13}C-NMR spectrum of **20** ($CDCl_3$, 100 MHz).

2. Mass spectra

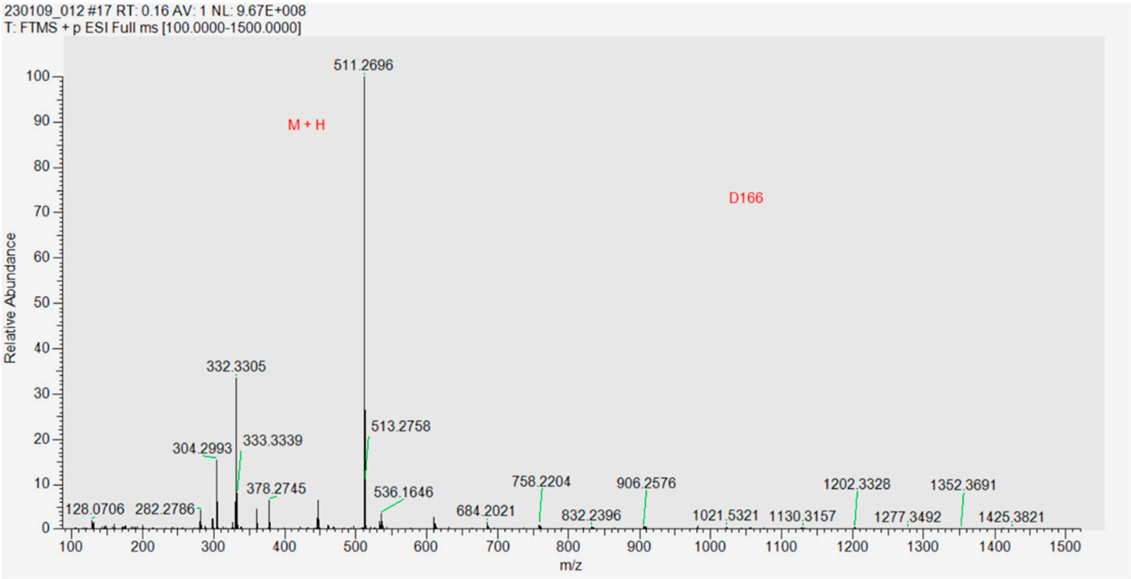

Figure S19 Mass spectrum of **7**.

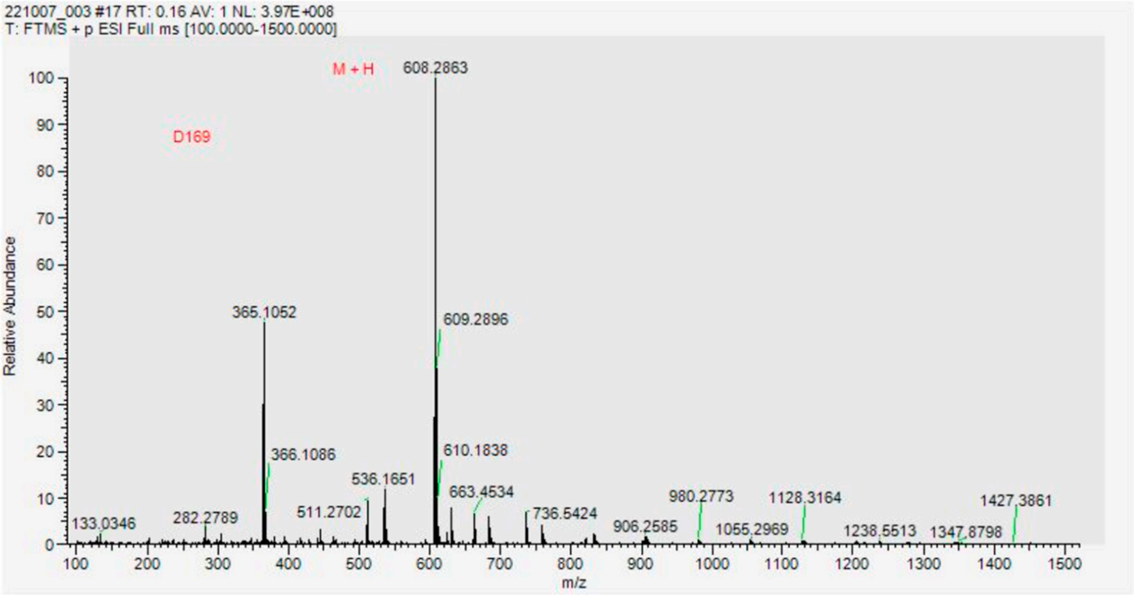

Figure S20 Mass spectrum of **9**.

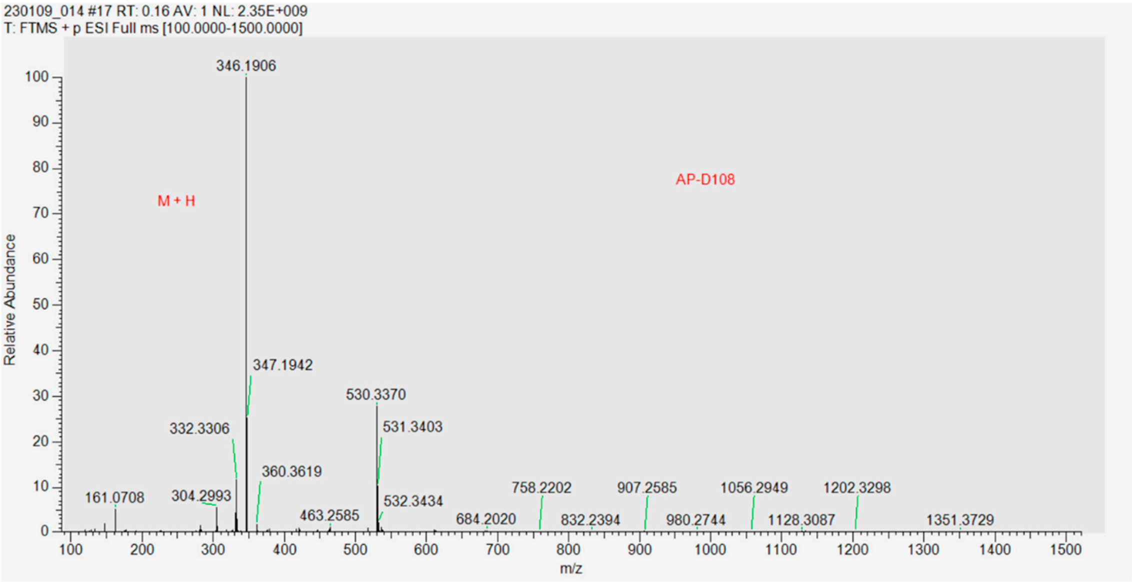

Figure S21 Mass spectrum of **11**.

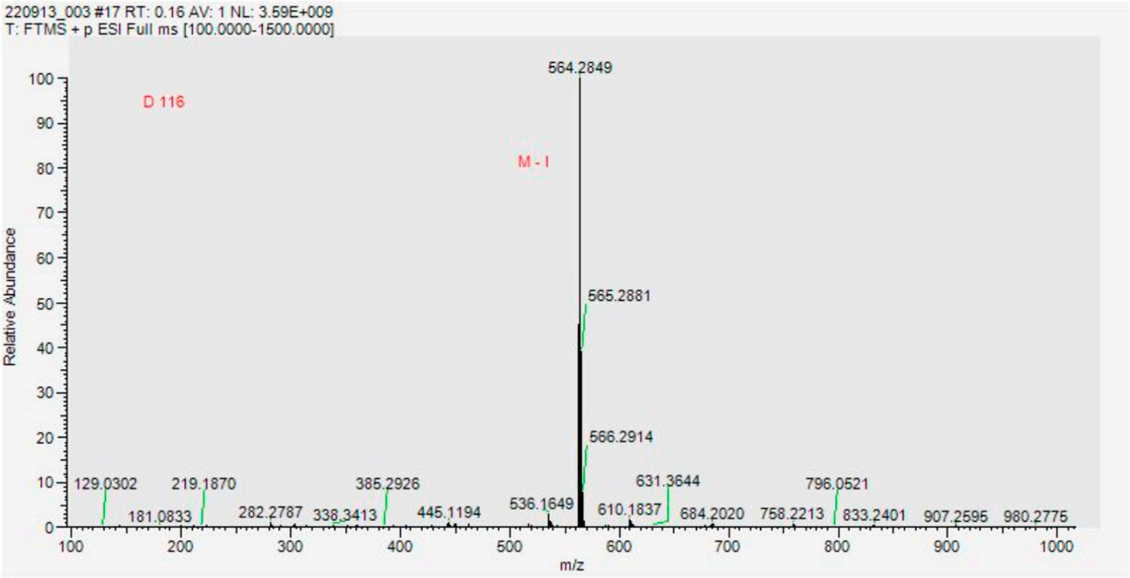

Figure S22 Mass spectrum of **12**.

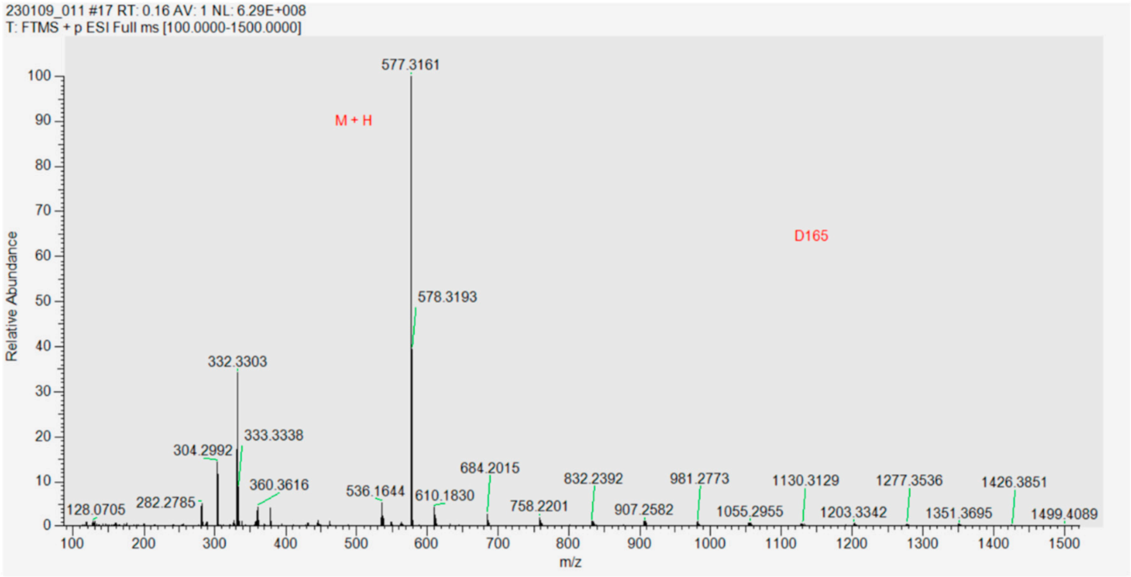

Figure S23 Mass spectrum of **13**.

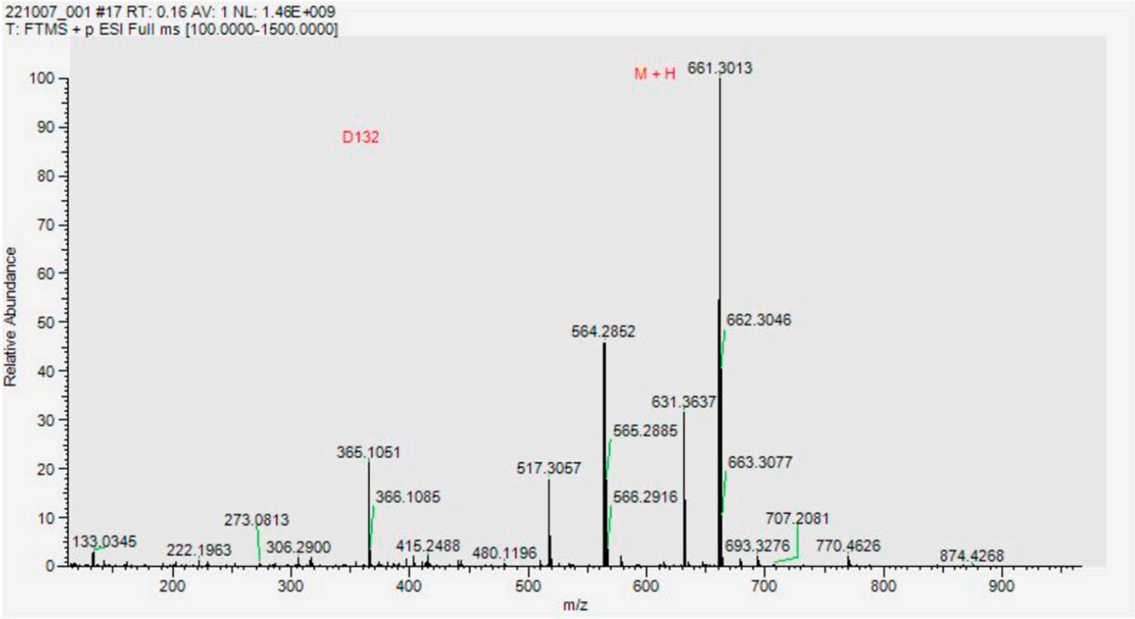

Figure S24 Mass spectrum of **14**.

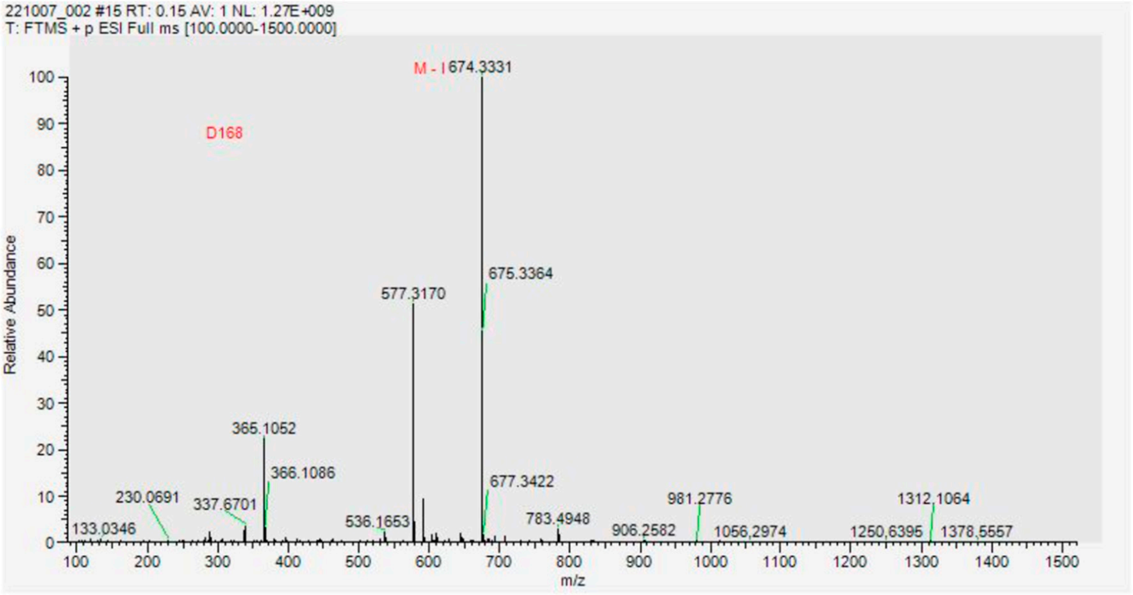

Figure S25 Mass spectrum of **15**.

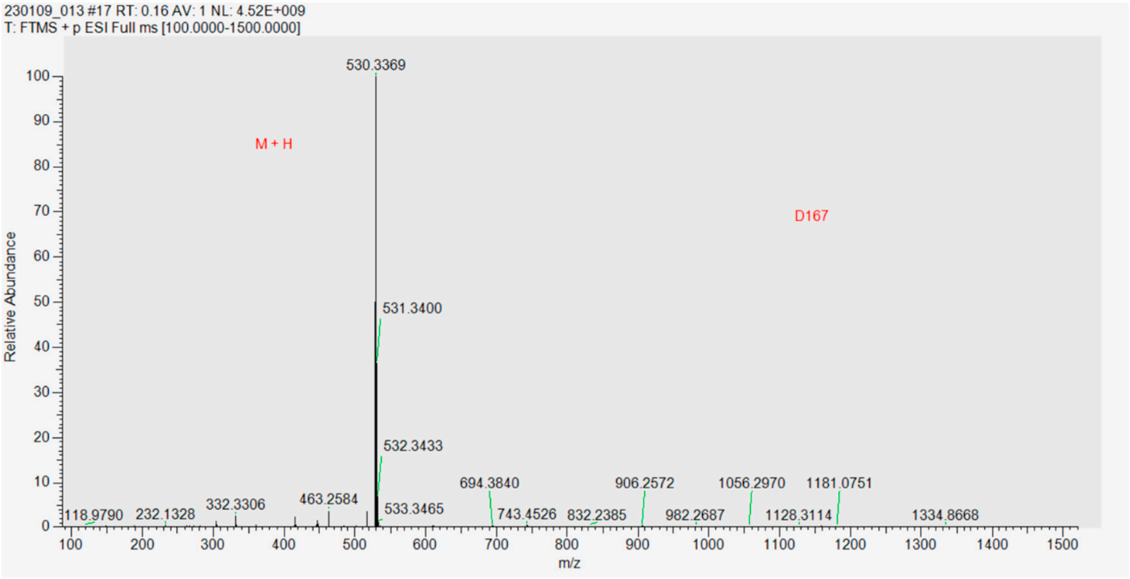

Figure S26 Mass spectrum of **18**.

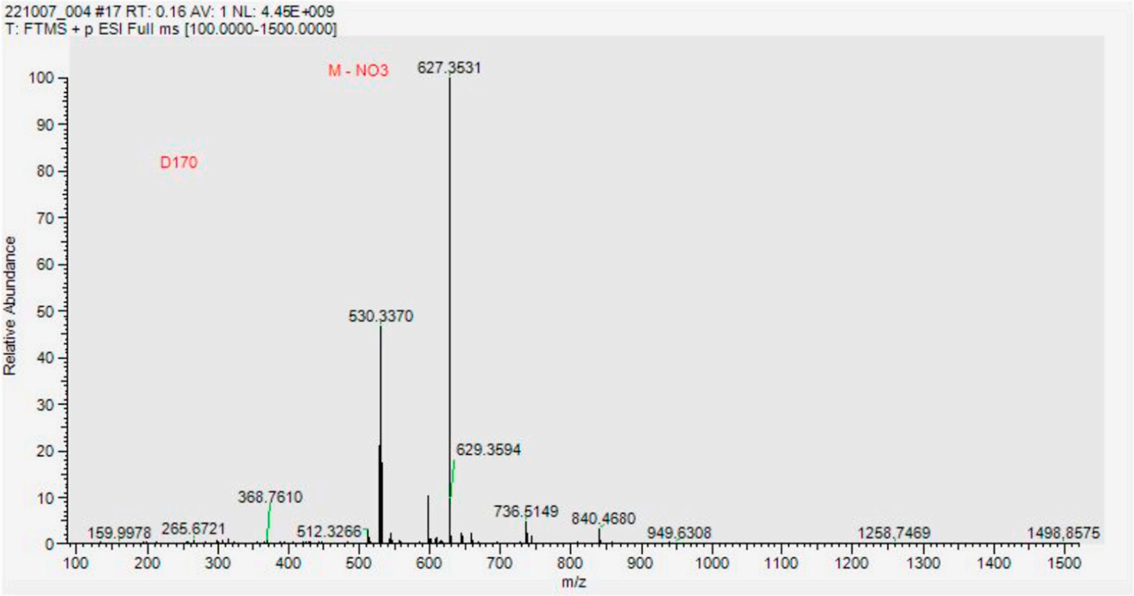

Figure S27 Mass spectrum of **20**.

3. Quantum chemical calculations

Table S1. Calculated absorption data of the lowest energy transition of selected derivatives.

**Compound**		Wavelength (nm)	f	Composition	Δ**r** (Å)
**8**	$S_0 \rightarrow S_1$	507.6	0.637	HOMO->LUMO (99%)	2.287
	$S_0 \rightarrow S_2$	455.9	1.352	H-1->LUMO (99%)	1.609
**9**	$S_0 \rightarrow S_1$	543.6	1.467	HOMO->LUMO (98%)	3.753
	$S_0 \rightarrow S_2$	495.2	0.709	H-1->LUMO (99%)	3.048
**14**	$S_0 \rightarrow S_1$	563.1	0.929	HOMO->LUMO (98%)	1.682
	$S_0 \rightarrow S_2$	485.5	1.372	H-1->LUMO (97%)	3.249
**15**	$S_0 \rightarrow S_1$	599.1	1.747	HOMO->LUMO (93%)	4.373
	$S_0 \rightarrow S_2$	535.6	0.689	H-1->LUMO (94%)	2.402
**19**	$S_0 \rightarrow S_1$	467.3	0.772	HOMO->LUMO (97%)	8.836
	$S_0 \rightarrow S_2$	434.8	1.166	H-1->LUMO (97%)	4.706
**20**	$S_0 \rightarrow S_1$	525.3	1.546	HOMO->LUMO (98%)	4.045
	$S_0 \rightarrow S_2$	456.8	0.518	H-1->LUMO (98%)	3.694

Table S2. Selected bond distances (Å), dihedral angle (°) and BLA (Å) of the compounds in the ground S_0 and excited S_1 states.

	r[10-18]		r[18-37]		r[37-39]		BLA		D[3-10-18-37]	
**Compound**	S_0	S_1	S_0	S_1	S_0	S_1	S_0	S_1	S_0	S_1
**8**	1.450	1.405	1.353	1.383	1.451	1.436	0.10	0.04	18.9	0.7
**9**	1.442	1.417	1.359	1.385	1.440	1.430	0.08	0.04	12.6	0.1
**14**	1.447	1.416	1.355	1.375	1.448	1.439	0.09	0.05	15.5	1.4
**15**	1.438	1.426	1.362	1.377	1.437	1.433	0.08	0.05	8.4	1.8
**19**	1.449	1.408	1.353	1.383	1.449	1.436	0.10	0.04	19.9	3.6
**20**	1.441	1.430	1.360	1.377	1.439	1.439	0.08	0.06	15.4	7.4

Table S3. Calculated emission data of the lowest energy transition of selected derivatives.

**Compound**		Wavelength (nm)	f	Composition	τ (ns)
**8**	$S_1 \rightarrow S_0$	576.2	0.546	HOMO->LUMO (99%)	9.11
**9**	$S_1 \rightarrow S_0$	583.2	1.543	HOMO->LUMO (99%)	3.30
**14**	$S_1 \rightarrow S_0$	602.8	0.877	HOMO->LUMO (98%)	6.21
**15**	$S_1 \rightarrow S_0$	623.0	1.850	HOMO->LUMO (95%)	3.14
**19**	$S_1 \rightarrow S_0$	522.7	0.702	HOMO->LUMO (98%)	5.83
**20**	$S_1 \rightarrow S_0$	560.2	1.678	HOMO->LUMO (99%)	2.80

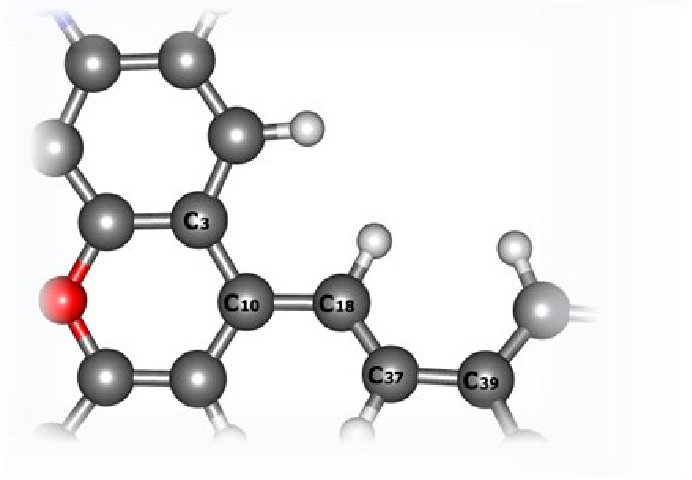

Figure S28 Numbering scheme adopted to all fluorescent dyes.
